# Effect of melatonin on cognitive function in adults with cognitive impairment: a multi-dimensional meta-analysis of randomized trials

**DOI:** 10.1186/s13195-025-01881-w

**Published:** 2025-11-03

**Authors:** Leona Yuen-Ling Leung, Hon-Lon Tam, Nestor Asiamah, Jonathan Ka-Ming Ho

**Affiliations:** 1 School of Nursing and Health Sciences, Hong Kong Metropolitan University, Hong Kong, China; 2 Hong Kong Nutrition Association, Hong Kong, China; 3 The Nethersole School of Nursing, The Chinese University of Hong Kong, Shatin, Hong Kong China; 4 Department of Health and Social Care, University of Essex, Colchester, UK

**Keywords:** Alzheimer's diseases, Dementia, Cognitive, Melatonin, Review

## Abstract

**Background:**

Cognitive impairment leads to poor daily social and occupational functions and sleep disturbances. Approximately two-thirds of all individuals with mild cognitive impairment (MCI) experience sleep problems that further reduce cognitive function. Melatonin, a hormone secreted by the pineal gland, has proven effective in mitigating sleep problems and cognitive function in individuals with MCI. The current review investigated the efficacy of melatonin in improving cognitive function in adults with cognitive impairment.

**Methods:**

Seven databases were systematically searched for relevant randomized controlled trials published (in English or Chinese) until April 2025. Two reviewers independently selected studies, assessed quality (using the Physiotherapy Evidence Database scale), and extracted data.

**Results:**

In total, 394 potentially eligible articles were identified. Finally, 8 studies (518 participants) were included. Five, one, and two studies had good, excellent, and low quality, respectively. Pooled results indicated that melatonin significantly improved cognitive function in adults with cognitive impairment (mean difference [MD]: 1.08; *p* < 0.0001). Subgroup analyses by treatment duration, administration time, and cognitive impairment level revealed that the effects of melatonin were significant when it was administered for 13–24 weeks (MD: 2.04; *p* < 0.00001), between the times of 20:30 and 21:00 (MD: 2.2; *p* < 0.00001), and to individuals with MCI (MD: 2.63; *p* < 0.000001).

**Conclusions:**

Our findings suggest that melatonin is relatively safe for individuals with cognitive impairment. Thus, we recommend it for adults with MCI. It should be administered between 20:30 and 21:00 for 13–24 weeks.

**Supplementary Information:**

The online version contains supplementary material available at 10.1186/s13195-025-01881-w.

## Background

Mild cognitive impairment (MCI) is an intermediate state between normal cognition and dementia [1, 2]. It is considered to be a transitional stage toward Alzheimer’s disease (AD) [3]. MCI can be further subtyped into amnestic and nonamnestic forms [1], with the amnestic subtype commonly progressing to AD [4]. Notably, individuals with dementia exhibit impaired daily social and occupational functions [2] and frequent sleep disturbances [5, 6].

A recent systematic review (SR) reported that the crude prevalence of MCI is 27%, whereas that of dementia is 7% [7]. In many individuals, MCI does not progress to dementia and may even revert to normal cognition [8]. However, approximately half of all individuals with MCI develop AD within 5 years [9]. Approximately 65% of all individuals with MCI experience sleep problems [10], which further impair their cognitive function [11].

Given the lack of effective treatment options for dementia, the MCI stage is a critical period in delaying disease progression [7]. Thus, early screening and intervention are crucial to preventing cognitive decline in individuals with MCI. Common tools used by health-care professionals to screen for dementia and evaluate cognitive function include the Mini-Mental State Examination (MMSE), Montreal Cognitive Assessment, and Mini-Cog [2].

Both pharmacological and nonpharmacological interventions are used to decelerate the progression of MCI to dementia. Nonpharmacological interventions include lifestyle modifications (e.g., cognitive training) and supplements aimed at improving cognitive function (e.g., Souvenaid) [12]. However, evidence on the effectiveness of lifestyle modifications remains inconclusive, and research indicates that Souvenaid does not confer substantial benefits in individuals with MCI [12]. Additionally, available pharmacological interventions, such as the use of acetylcholinesterase inhibitors, anti-inflammatory agents, and nootropics, are focused on mitigating symptoms rather than reversing the condition [12]. Furthermore, because of their health risks like increased risk of fall and dependance, sedatives such as benzodiazepines are rarely used to manage sleep problems in individuals with MCI or dementia [11].

Melatonin, a hormone secreted by the pineal gland, can regulate the circadian rhythm, exert antioxidative effects, and modulate immune responses [13]. It has been considered as a dietary supplement in the United States and is available over-the-counter without a prescription since 1994 [14]. An analysis of UK Biobank data (*N* = 479,420) revealed that adequate sleep duration is associated with improved cognitive performance [15]. Therefore, melatonin supplementation is considered a potential intervention for managing sleep problems and improving cognitive function in individuals with MCI or dementia [13]. However, no SR has comprehensively analyzed the beneficial effects of melatonin against cognitive impairment across different dimensions such as administration time, level of cognitive impairment, and dementia severity. In this review, we analyzed the efficacy of melatonin in improving cognitive function in adults with cognitive impairment. Efficacy was analyzed with consideration of the duration of treatment, dosage of treatment, type and level of cognitive impairment, and time of melatonin administration.

## Methods

### Guidelines

The protocol for this SR and meta-analysis was registered with the International Prospective Register of Systematic Reviews (PROSPERO) (registration number: CRD42024554855). This study adhered to the 2020 Preferred Reporting Items for Systematic Reviews and Meta-Analyses guidelines [16]

### Review question

Can melatonin effectively improve cognitive function in adults with cognitive impairment? To answer the question, we performed subgroup analyses by treatment duration and dosage, type and level of cognitive impairment, dementia severity, and melatonin administration time.

### Eligibility criteria

This SR included randomized controlled trials where adults (age ≥ 18 years) with any types of cognitive impairment, such as MCI and any forms of dementia (population), received supplementary melatonin (intervention) or placebo (comparator) and underwent assessments of postintervention changes in cognitive function (outcome). Articles published in English or Chinese were eligible for inclusion.

We excluded studies focusing on animals, children, or adolescents; those involving multiple interventions (i.e., melatonin supplementation plus other interventions); and those analyzing pharmacodynamics or pharmacokinetics.

### Search strategy

A three-step approach was adopted to identify potentially eligible articles. The Cochrane Library (Cochrane Central Register of Controlled Trials and Cochrane Methodology Register), China National Knowledge Infrastructure, CINAHL, SCOPUS, MEDLINE, Embase, and Web of Science databases were systematically searched for relevant articles published until April 2025. First, MEDLINE was searched to identify the keywords in the titles or abstracts and index terms of potentially eligible studies. Second, all electronic databases were extensively searched using the identified keywords and Medical Subject Headings terms—such as “melatonin,” “cognition,” “cognitive,” “cognitive function,” “dementia,” and “Alzheimer.” Third, other literature sources were manually searched to identify eligible original studies. In addition, the reference lists of all identified studies were reviewed for additional original studies. Furthermore, the ClinicalTrial.gov registry was searched for relevant and ongoing studies. The search strategies used for the seven databases are presented in Supplementary S-Table 1.

### Article selection

The search results were imported into a Web and mobile application, Rayyan, for duplicate removal [17]. A pilot screening and selection of the studies was conducted to ensure consistency before reviewing all studies. Two reviewers (LYL and HL) independently screened the titles and abstracts of all identified studies to confirm their eligibility for inclusion in this SR. If a study was deemed eligible for inclusion or if the information provided in the title or abstract was insufficient, the full text was reviewed for eligibility assessment. Between-reviewer disagreements were resolved through discussion with a third reviewer (JKM). The agreement level of the reviewers was assessed using Cohen’s Kappa coefficient, with the result *K* = 0.8160 (excellent agreement) [18].

### Study quality assessment

Before the inclusion of eligible studies, LYL and HL independently assessed methodological quality by using the Physiotherapy Evidence Database (PEDro) scale [19]. This scale comprises 11 yes/no items. With the exception of the first, all items contribute 1 point each to the total score, which ranges from 0 to 10 points. The included studies were rated as “excellent” (9–10 points), “good” (6–8 points), “fair” (4–5 points), or “poor” (< 4 points). Risk of bias was evaluated in the following domains: generation of a randomization sequence, concealment of treatment allocation, blinding of participants and personnel, completeness of outcome data, selective reporting of outcome data, and other sources of bias (Supplementary S-Table 2). Between-reviewer disagreements were resolved through discussion with JKM.

### Data extraction

LYL and HL independently extracted the following data from the included studies by using a self-developed extraction form (Microsoft Excel): author name; publication year; study aim, design, country, setting, and duration; sample size; population characteristics; interventions; and outcome measures. Authors of the original articles were contacted for data that were missing or could not be extracted. JKM confirmed the accuracy of the extracted data.

### Data synthesis and statistical analysis

If three or more studies reported the outcome of interest, data were pooled and subjected to meta-analysis, which was performed using Review Manager [RevMan], [Computer program], Version 5.4, The Cochrane Collaboration, 2020. Mean difference (MD) values and corresponding 95% confidence intervals (CIs) were calculated for continuous data collected using the same scale. Heterogeneity across the included studies was assessed using the *I²* statistic, with *I²* values of 25%, 50%, and 75% indicating low, moderate, and high levels of heterogeneity, respectively [20, 21]. In cases of low to moderate heterogeneity or when only a few studies were available to obtain an accurate estimate of the between-studies variance, a fixed-effects model was used; otherwise, a random-effects model was adopted [22, 23]. Sensitivity analysis was performed for meta-analysis results with high heterogeneity or when the eligibility of certain studies was doubtful. For outcomes reported in a single SR or when data pooling was statistically infeasible, the findings were summarized narratively. If sufficient data were available, subgroup analyses by treatment duration and dosage, different types and levels of cognitive impairment, dementia severity, and melatonin administration time were performed.

### Protocol deviation

This review strictly adhered to the registered protocol, with the exception that the criteria for model selection in the meta-analyses were refined during the review process.

### Patient and public involvement

Neither patients nor the public was involved in the conduct of this study or the preparation of the manuscript.

## Results

### Study selection

**Fig. 1:**
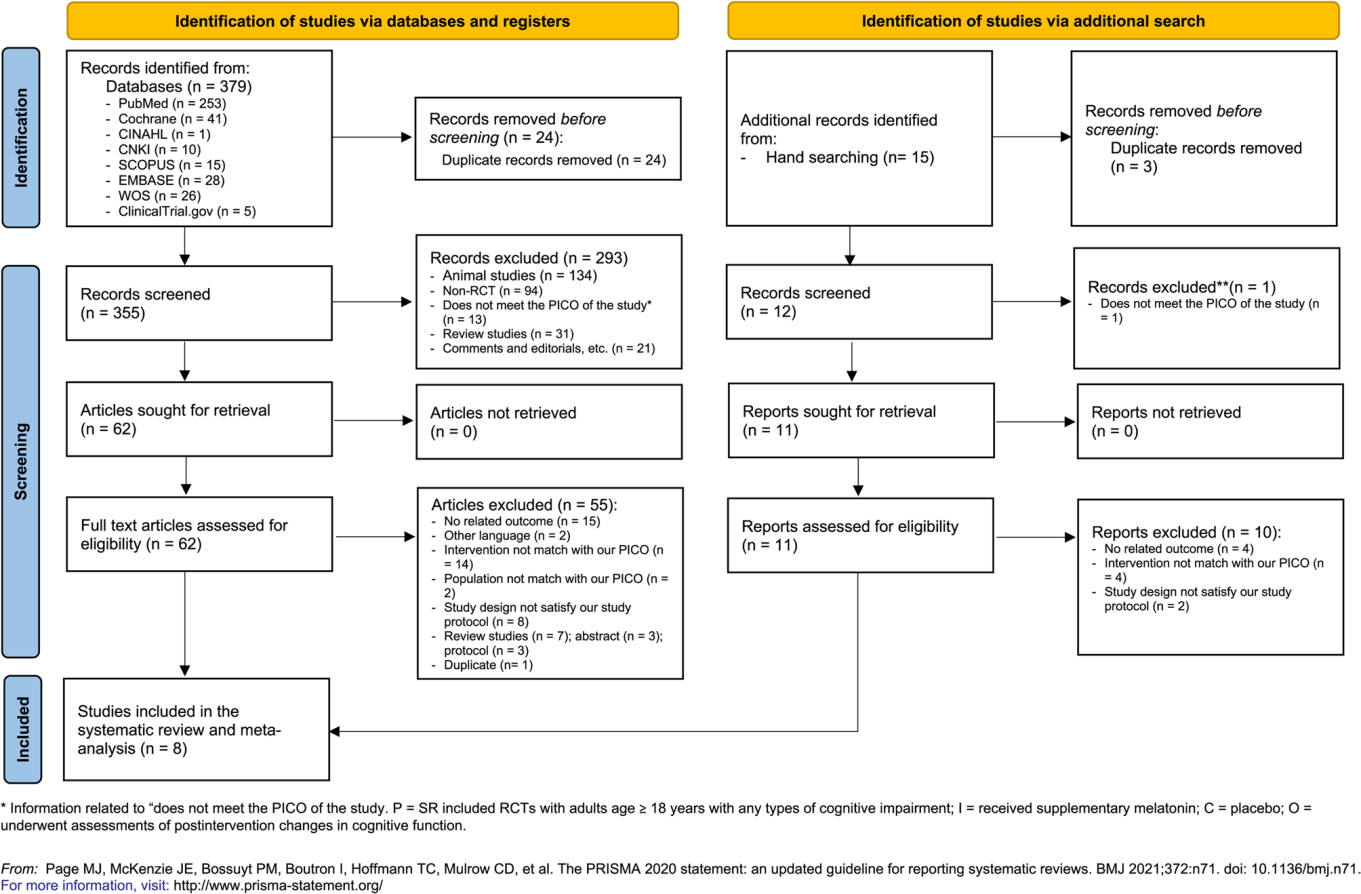
Flow diagram of retrieving and selecting studies

The literature search yielded 394 English and Chinese articles. After the removal of 27 duplicate records and 294 irrelevant articles, 73 articles were subjected to full-text screening. From these articles, 65 were excluded due to irrelevant populations, interventions, outcomes, or study designs; duplicate entries; articles available only as abstracts or protocols; and those not published in English or Chinese. The excluded studies are listed in Supplementary S-Table 3. Finally, eight studies (518 participants) were included in this SR (Fig. 1) [5, 6, 11, 24–28].

### Study characteristics

Table 1 presents the characteristics of the included studies. The studies were published between 2002 and 2024. Two studies were conducted in China [25, 28], whereas the remaining studies were conducted in Australia [26], Japan [24], Mexico [11], the Netherlands [27], the United States [5], and the United Kingdom plus the United States [6]. Sample sizes ranged from 20 [24] to 157 [5]. Daily melatonin dosages ranged from 2 mg [6] to 25 mg [26]. Intervention durations ranged from 5 weeks [24] to 3.5 years [27]. Participants included patients with AD [6, 24, 25], dementia [27], MCI [26, 28], or AD with sleep disturbances [5]. Administration times for melatonin were as follows: 20:30 [24, 28], 21:00 [11], before bedtime [27], and 1 to 2 h before bedtime [5, 6, 26]. Notably, Gao et al. did not report the time of melatonin administration [25]. All included studies did not report any industry sponsorship or funding. Outcome measures included cognitive function, dementia severity, depression severity, functional independence, sleep quality, self-esteem, and functional impairment.

**Table 1:** Review characteristics of included studies (*n* = 8)

Author (Year)	Design, Country, no. study site	Number of participants (% female)	Mean age in years ± SD or Mean age in years (range)	Study population	Primary Aim	Intervention	Control	Study duration	Outcomes	Melatonin Administration Time	Attrition Rate (%)	ITT	Protocol	Grant support (GS)
Asayama (2003) [24]	Parallel RCT, Japan, one	20 (85%)	79.2 ± 6.4	Alzheimer’s dementia (AD)	Evaluate melatonin effects on the disturbance of sleep-wake rhythm in the night and daytime, cognitive and non-cognitive function	Melatonin 3 mg/daily	Placebo	5-week	Clinical dementia rating scale (CDR), Mini Mental State Examination (MMSE), Alzheimer’s Disease Assessment Scale-cognition (ADAS-Cog)	20:30–21:00	0%	N	N/A	N/A
Gao (2009) [25]	Parallel RCT, China, one	32 (N/A)	77 ± 2.9	AD	Investigate the effect of melatonin on mild AD	Melatonin 2.9 mg/daily	Placebo	24-week	MMSE	At night time	3.125%	N/A	N/A	N/A
Menczel Schrire (2024) [26]	Parallel RCT, Australia, one	40 (48%)	Melatonin: 66.6 ± 1.2 placebo: 69.6 ± 7.4	Mild cognitive impairment (MCI) ≤ 33 Memory and Ageing Telephone Screen	Provide feasibility, acceptability and tolerability data, as well as preliminary effect size data on potential primary and secondary outcomes, to inform a larger definitive trial	Melatonin 25 mg/daily	Placebo	12-week	Cambridge Neuropsychological Test Automated Battery	1–2 h before bedtime	5%	Y	Y	Y
Morales-delgado (2018) [11]	Parallel RCT, Mexico, one	40 (77.42%)	Melatonin: 82.2 ± 5.8 placebo: 83.1 ± 7.4	Aged 65 or above, mild or moderate dementia	Evaluate the effect of melatonin over sleep quality in patients with dementia	Melatonin 5 mg/daily	Placebo	8-week	MMSE	20:30–21:00	20%	N	Y	Y
Riemersma-van der Lek (2008) [27]	RCT (2 × 2 factorial design), Netherlands, twelve	91(85.72%)	Melatonin: 85 ± 5 placebo: 86 ± 5	Dementia	Evaluate the effectiveness of daily supplementation of light and/or melatonin on cognitive, mood, behavioral, functional, and sleep disturbance	Melatonin 2.5 mg/daily	Placebo	3.5-year	MMSE	Immediately before bedtime	79.12%	Y	Y	Y
Singer (2003) [5]	3-arm Parallel RCT, USA, multi-center	157 (N/A)	77.4 ± 8.9	Diagnosis of probable AD, a nighttime sleep disturbance	Determine the safety and efficacy of 2 dose formulations of melatonin for the treatment of insomnia in patients with AD	Melatonin sustained-release (SR) 10 mg/daily Melatonin (SR) 2.5 mg/daily	Placebo	8-week	MMSE, ADAS-Cog, ADCS-ADL inventory	1–2 h before bedtime	6.37%	Y	Y	Y
Wade (2014) [6]	Single blind run-in, Parallel RCT, UK and USA, five centers	60 (N/A)	Prolonged-released melatonin (PRM): 73.5 ± 8.6, placebo: 77.3 ± 6.6 (52–85-yr)	Mild to moderate AD and MMSE score of ≥ 15	Evaluate the effects of add-on PRM versus placebo on cognition and sleep, in patients with mild to moderate AD who are treated with standard AD therapy (acetylcholinesterase inhibitors, with and without memantine)	PRM (Circadin^®^ 2 mg)	Placebo	28-week (24 weeks intervention period and a 2-week placebo run-out period)	ADAS-Cog, MMSE	1–2 h before bedtime	16.44%	N	Y	Y
Xu (2020) [28]	Parallel RCT, China, one	79 (51.89%)	66.7 ± 8.6	MCI (MMSE: 24–30 points)	Investigate the melatonin induced effects on the lamina cribrosa thickness of patients with mild cognitive impairment	Melatonin 0.15 mg/kg daily	Placebo	6-month	MMSE	20:30–21:00	0%	N	Y	N/A

### Study quality

The methodological quality of the included studies was assessed using the PEDro scale (Supplementary S-Table 4). Quality scores ranged from 5 to 10, with an average of 7.375. Two studies had fair quality [25, 28], five had good quality [5, 6, 11, 24, 26], one had excellent quality [27]. All studies reported random allocation (Item 2 of the PEDro scale). However, only one study adopted the intention-to-treat approach [27], and only three studies reported therapist (intervention provider) blinding [6, 11, 27].

### Results of meta-analysis

In addition to performing a meta-analysis of cognitive function, we performed subgroup analyses and the results as follow.

#### Overall effects of melatonin on cognitive function

**Fig. 2:**
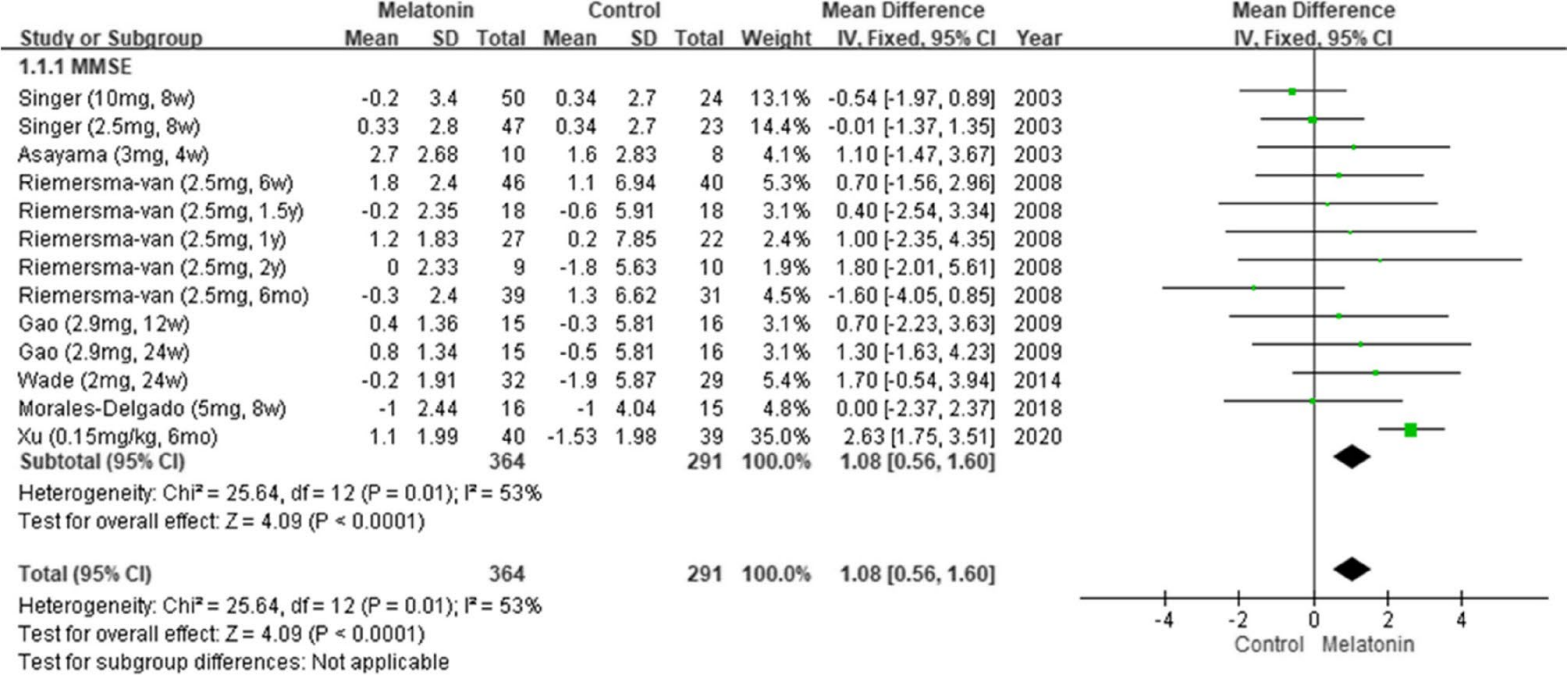
Changes in cognitive functions (MMSE)

**Fig. 3:**
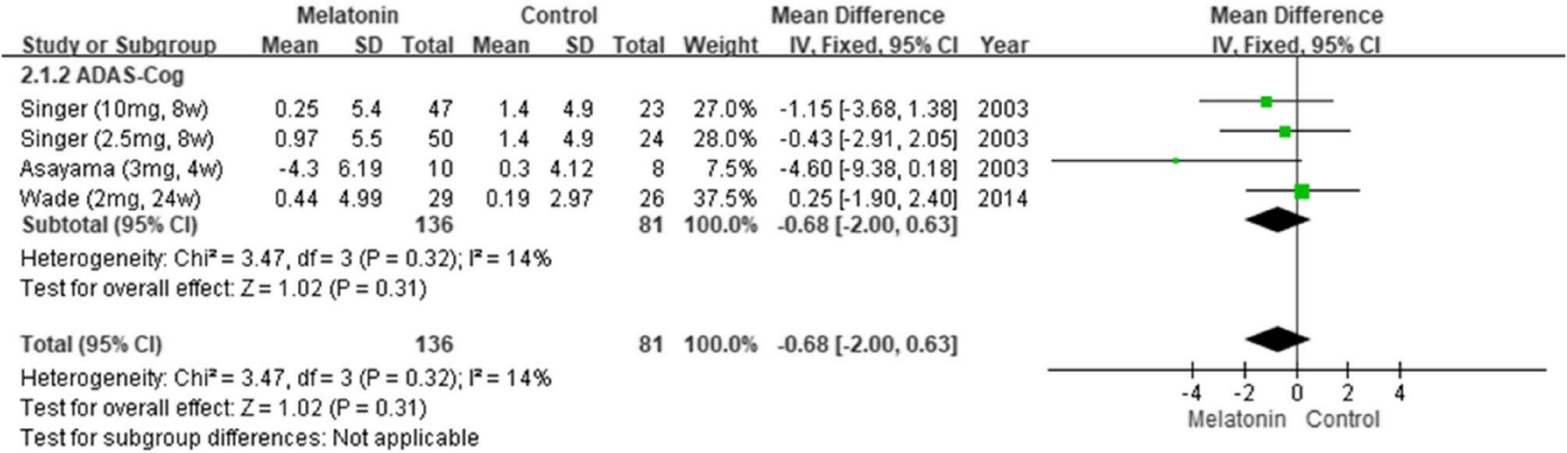
Changes in cognitive functions (ADAS-Cog)Changes in cognitive functions (ADAS-Cog)

The pooled results from seven studies indicated that melatonin supplementation led to significant improvements in cognitive function [5, 6, 11, 24, 25, 27, 28], as indicated by the patients’ MMSE scores (MD: 1.08; 95% CI: 0.56–1.60; *I*^*2*^ = 53%; *p* < 0.0001; Fig. 2). However, their Alzheimer’s Disease Assessment Scale–Cognitive Subscale (ADAS-Cog) scores revealed no significant improvement (MD: −0.68; 95% CI: −2.00 to 0.63; *I*^*2*^ = 14%; *p* = 0.31; Fig. 3). Consistently, Menczel Schrire et al. reported that melatonin did not improve cognitive function [26], as indicated by the patients’ Cambridge Neuropsychological Test Automated Battery scores (MD: −0.06; 95% CI: −0.50 to 0.39) of the subscale Total Errors Adjusted, Partial Assessment Learning Task.

#### Results of subgroup analysis by treatment duration

**Fig. 4:**
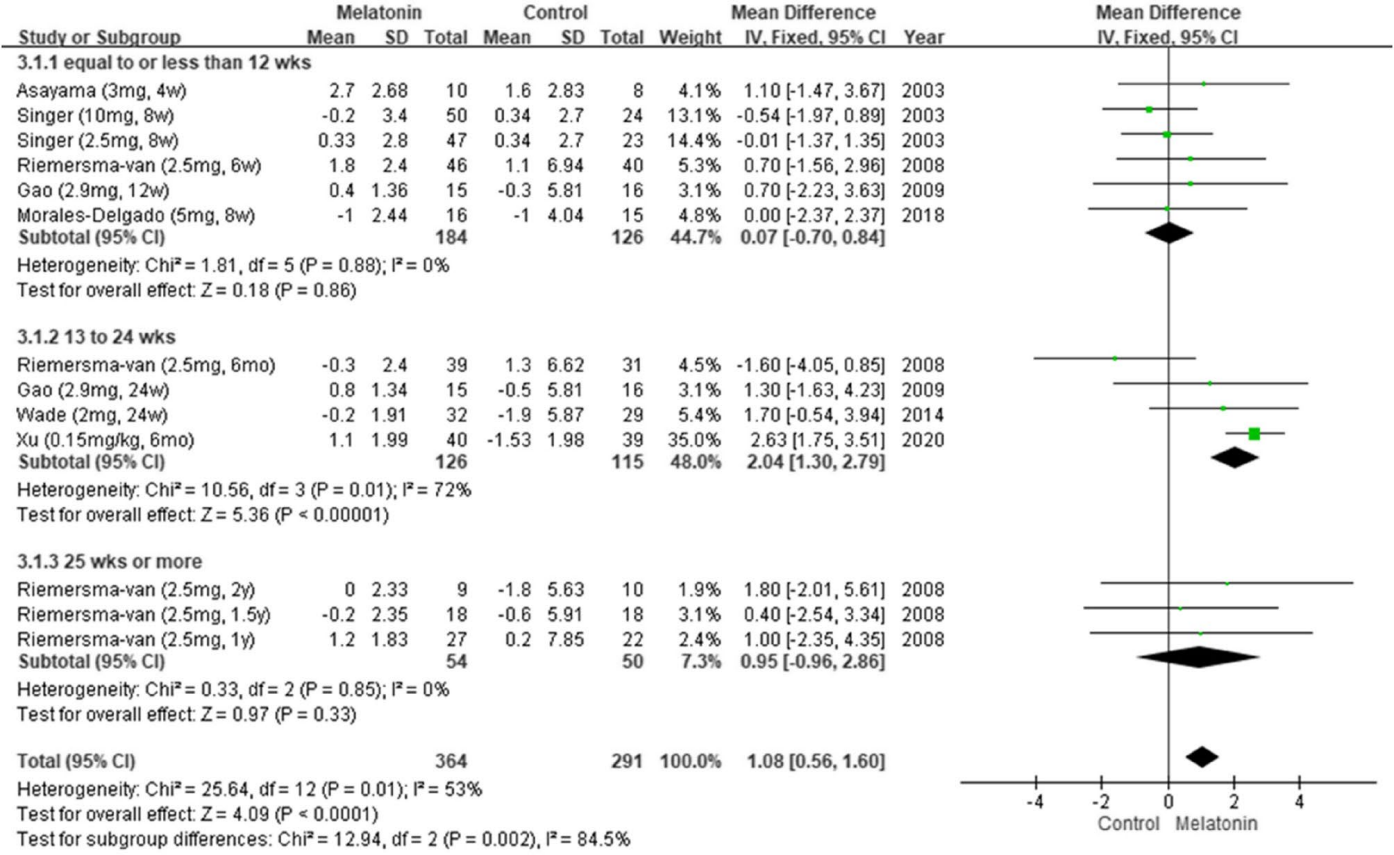
Changes in cognitive functions in different durations (MMSE)

**Fig. 5:**
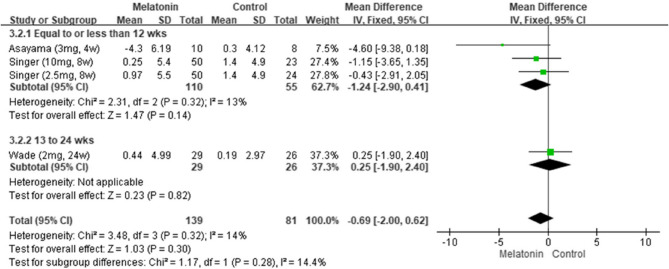
Changes in cognitive functions in different durations (ADASCog)

We performed a subgroup analysis by treatment duration. Pooled data from seven studies [5, 6, 11, 24, 25, 27, 28] suggested that melatonin significantly improved cognitive function when administered for 13–24 weeks (MD: 2.04; 95% CI: 1.30–2.79; *I*^*2*^ = 72%; *p* < 0.00001; Fig. 4), as indicated by the patients’ MMSE scores. However, pooled data from three studies revealed no significant improvement for any treatment duration, as indicated by the patients’ ADAS-Cog scores (Fig. 5) [5, 6, 24].

#### Results of subgroup analysis by treatment dosage

**Fig. 6:**
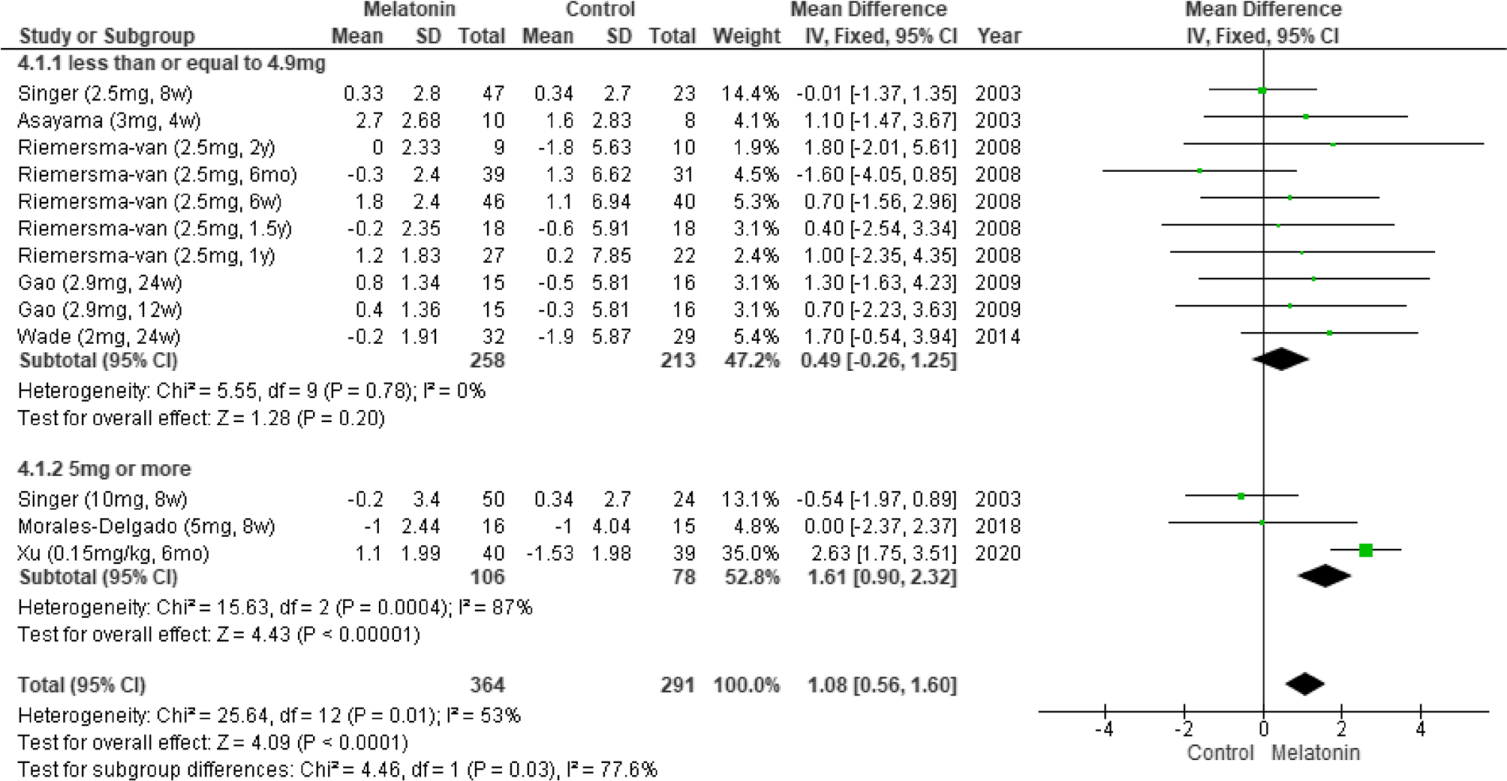
Changes in cognitive functions at different dosages (MMSE)

**Fig. 7:**
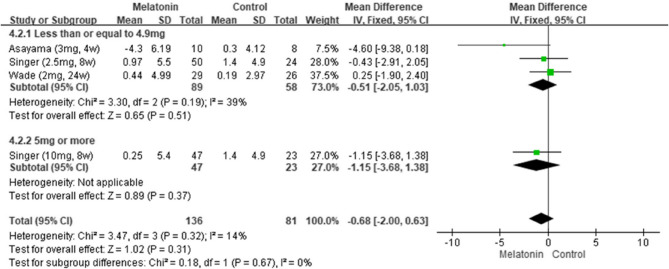
Changes in cognitive functions at different dosages (ADASCog)

We performed a subgroup analysis by treatment dosage. Pooled data from seven studies revealed that melatonin significantly improved cognitive function when administered at a daily dosage of ≥ 5 mg (Fig. 6) [5, 6, 11, 24, 25, 27, 28], as indicated by the patients’ MMSE scores. However, cognitive outcomes for a daily dosage of ≥ 5 mg exhibited high heterogeneity (MD: 1.61; 95% CI: 0.90 to 2.32; *I*^*2*^ = 87%; *p* < 0.00001; Fig. 6). Pooled data from three studies suggested no significant effect of any dosage, as indicated by the patients’ ADAS-Cog scores (Fig. 7) [5, 6, 24].

#### Results of subgroup analysis by cognitive impairment type

**Fig. 8:**
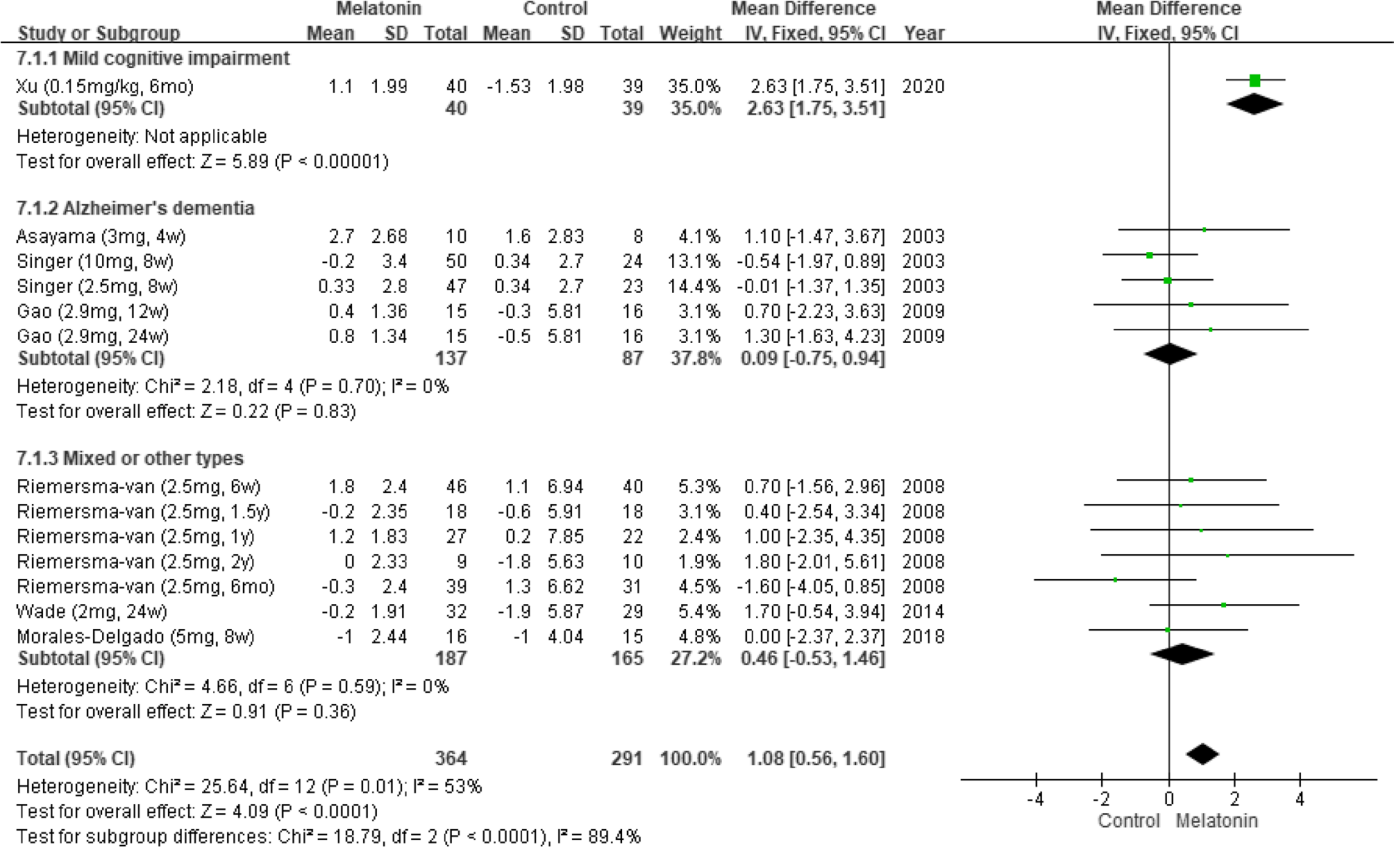
Changes in cognitive functions across different types of cognitive impairment (MMSE)

We performed a subgroup analysis by cognitive impairment type. Cognitive impairment was categorized as AD [5, 6, 25], MCI [28], or mixed/other types (e.g., mild or moderate dementia) [6, 11, 27]. The meta-analysis indicated that melatonin exerted no significant effect on cognitive function in individuals with AD or mixed/other types of cognitive impairment. However, Xu et al. reported that melatonin significantly improved cognitive function in individuals with MCI (MD: 2.63; 95% CI: 1.75–3.51; *p* < 0.00001; Fig. 8) [28].

#### Results of subgroup analysis by dementia severity

**Fig. 9:**
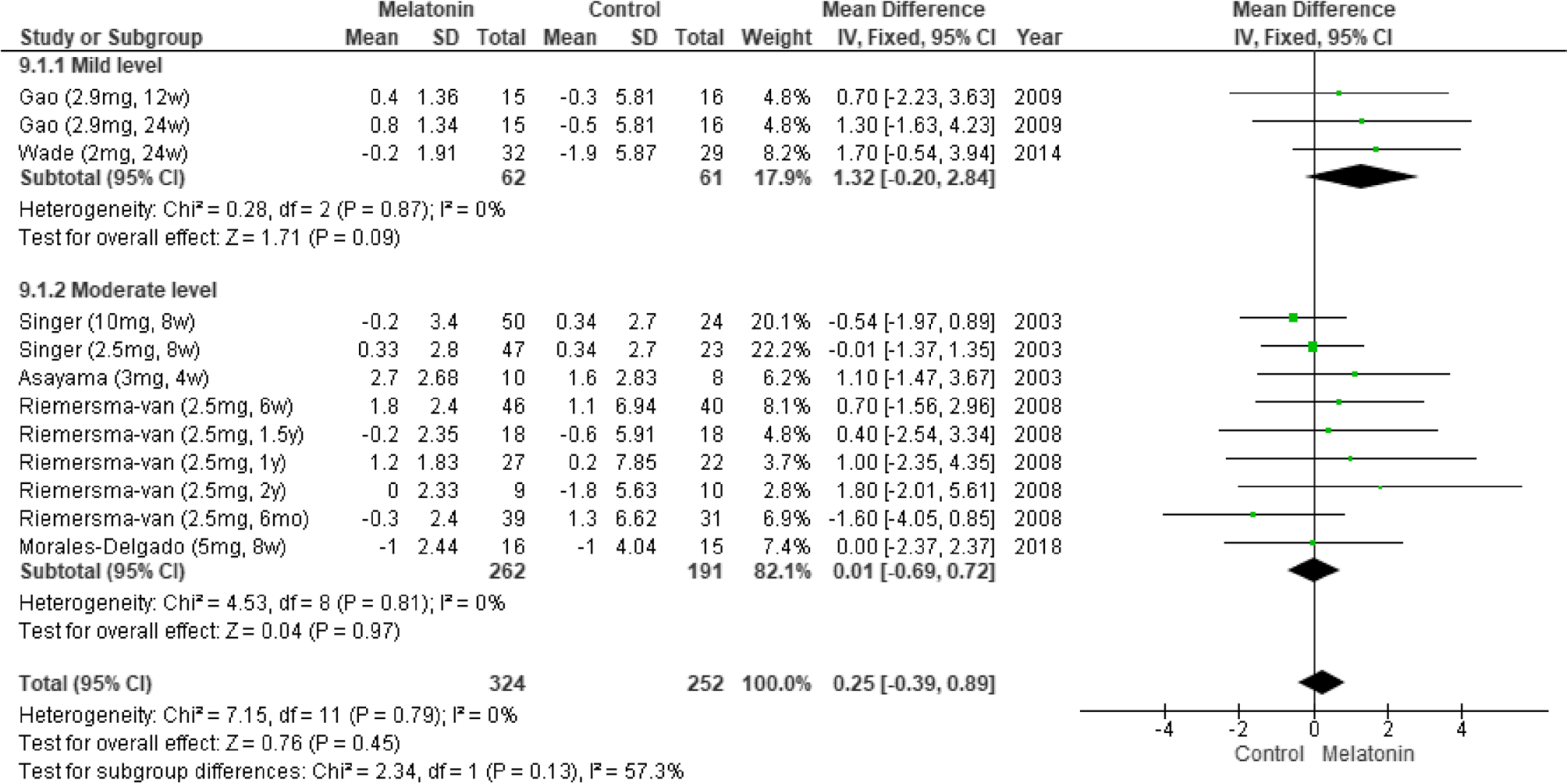
Changes in cognitive functions across different levels of dementia (MMSE)

We performed a subgroup analysis by dementia severity. On the basis of the MMSE score, dementia was categorized as mild [6, 25] or moderate [5, 11, 24, 27]. The pooled results indicated that melatonin insignificantly improved cognitive function in individuals with mild dementia (MD: 1.32; 95% CI: −0.20–2.84; *I*^*2*^ = 0%; *p* = 0.09; Fig. 9).

#### Results of subgroup analysis by melatonin administration time

**Fig. 10:**
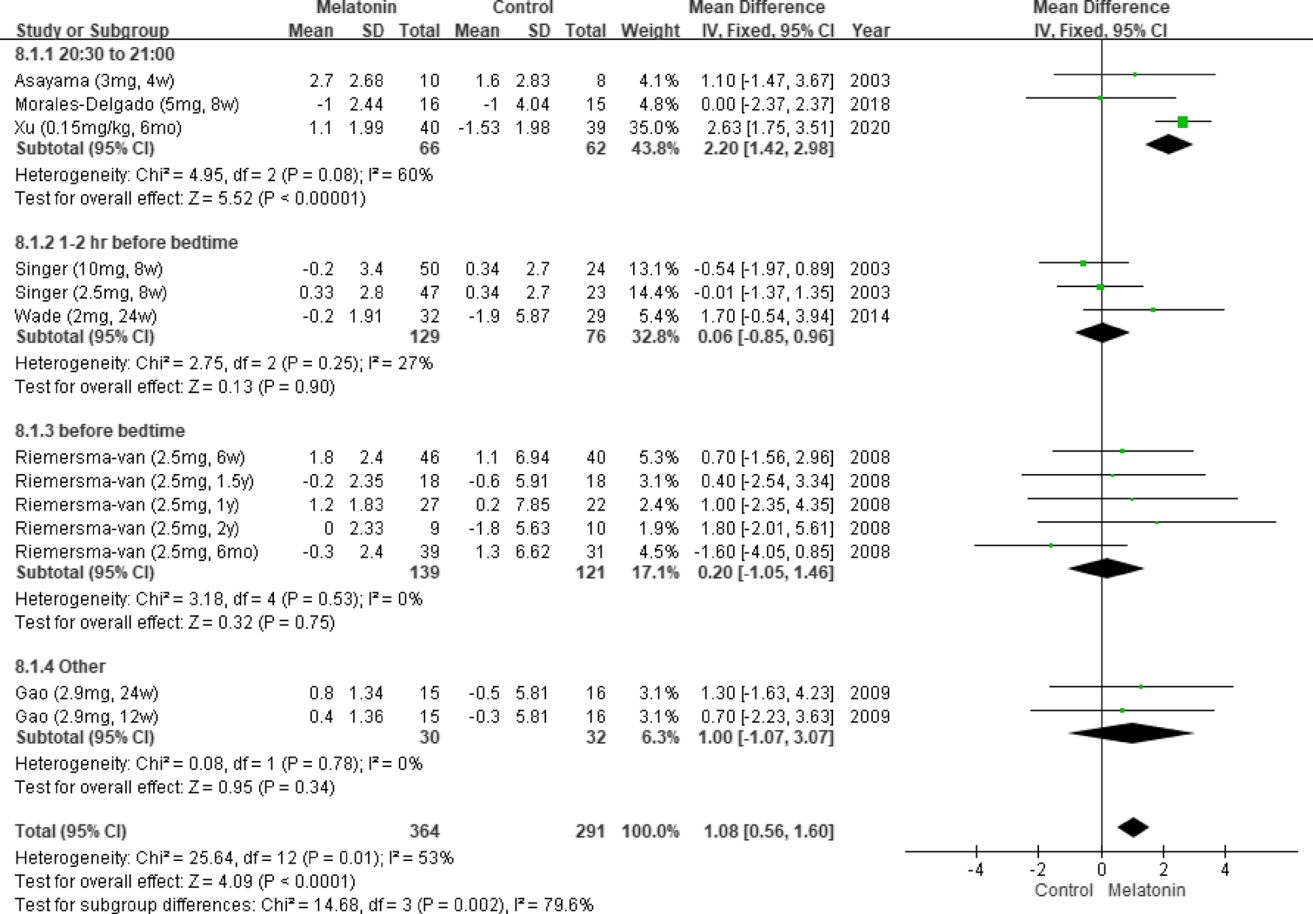
Changes in cognitive functions at different administration times (MMSE). Remarks: Other = at night time, not specify the administration time of melatonin

We performed a subgroup analysis by melatonin administration time. The drug was administered between 20:30 and 21:00 [11, 24], 1 to 2 h before bedtime [5, 6, 26], immediately before bedtime [27], or at other times [25]. The results indicated that melatonin significantly improved cognitive function when administered between 20:30 and 21:00 (MD: 2.20; 95% CI: 1.42–2.98; *I*^*2*^ = 60%; *p* < 0.00001; Fig. 10).

#### Results of sensitivity analysis by treatment dosage

Sensitivity analysis was performed to investigate the effect of a daily melatonin dosage of ≥ 5 mg on cognitive function. After the exclusion of the study by Singer et al. [5], the *I*^*2*^ value for heterogeneity remained high (MD: 2.31; 95% CI: 1.49–3.14; *I*^*2*^ = 76%; *p* < 0.00001; Supplementary S-Table 5). When the study by Xu et al. was excluded [28], both the *I*^*2*^ value for the subgroup analysis and the overall *I*^*2*^ value decreased to 0%, indicating the elimination of heterogeneity. However, the results regarding the effect of the indicated melatonin dosage on cognitive function became nonsignificant (Supplementary S-Table 5).

### Adverse effects

Two studies reported no adverse effects (AEs) of the intervention [24, 28]. Two other studies reported no AEs throughout the study period [11, 25]. The remaining four studies reported AEs separately for the intervention and control groups [5, 6, 26, 27]. The AEs of the intervention groups included gastrointestinal distress, fall, abnormal dreams, etc., where the AEs of the control groups included fatigue, insomnia, sinusitis, etc. (Supplementary S-Table 6).

### Attrition rate

All studies reported attrition rates, which ranged from 0% [24, 28] to 96.7% [27]. The high attrition rate in the study by Lek et al. occurred during a 3.5-year follow-up period [27]. However, this high rate did not influence treatment outcomes when the analysis was limited to the first 1.5 years of follow-up. Only one study reported an overall attrition rate without differentiating between the study groups [25].

## Discussion

### Summary of evidence

To the best of our knowledge, this is the first SR and meta-analysis to analyze the efficacy of melatonin in improving cognitive function in adults with cognitive impairment and the effects of treatment duration and dosage, type and level of cognitive impairment, dementia severity, and administration time on this efficacy. Data from eight studies were analyzed [5, 6, 11, 24–28]. The pooled results revealed that melatonin significantly improved cognitive function, as indicated by patients’ MMSE scores. This finding differs from those of Wang et al. [29] and Xu et al. [30], who reported no significant improvement on the basis of patients’ MMSE or ADAS-Cog scores.

In this study, subgroup analyses indicated that melatonin significantly improved cognitive function when administered for 13–24 weeks, between the times of 20:30 and 21:00, and to individuals with MCI. Similarly, Gao et al. reported that melatonin supplementation for 24 weeks markedly improved cognitive function in individuals with early AD [25], as indicated by their MMSE scores. The researchers suggested that long-term melatonin use can decelerate the progression of AD in its early stages. These findings align with those of Sumsuzzman et al. [13], who concluded that melatonin is relatively effective in individuals with MCI and when administered for >12 weeks, as indicated by patients’ MMSE but not their ADAS-Cog scores.

Our findings are in line with a previous review that some outcomes assessed by MMSE demonstrated significant results, but all outcomes assessed with ADAS-Cog showed no significant differences [13]. In comparison with ADAS-Cog, MMSE is more sensitive to measuring changes in MCI and early stages of AD [31]. Another possible reason is related to the focus of the scales that ADAS-Cog emphasizes ideation and motor planning, while MMSE focuses on attention [32].

Although a high attrition rate (96.7%) was noted in the reviewed literature, it occurred in a single study with a follow-up duration of 3.5 years [27]. The main reasons for attrition were the closure of facilities, high vulnerability of the population, and admission to nursing homes [27]. However, attrition rates in the remaining studies ranged from 0% to 20%, which is considered acceptable [33].

### Reporting biases

All included studies had fair [25, 28] to excellent [27] quality. All studies were double-blinded but lacked blinding of all therapists (Item 7 of the PEDro scale). Xu et al. reported that one outcome assessor was blinded to group allocation and clinical diagnosis [28]. The lack of assessor blinding may have led to overestimation of intervention effects [34]. Six studies (75%) did not adopt the intention-to-treat approach (Item 9 of the PEDro scale) [5, 6, 11, 24, 25, 28], which increases the limitations and reduces the accuracy of the findings [35, 36].

### Strengths

This study has several strengths. First, to the best of our knowledge, it is the first study to evaluate the efficacy of melatonin in improving cognitive function in adults with cognitive impairment and the effects of treatment duration and dosage, cognitive impairment type, dementia severity, and administration time on this efficacy. Second, the quality of the included studies was assessed using the well-validated PEDro instrument. Third, the SR protocol was registered with the PROSPERO. No amendments were made after registration, which increased the transparency of the review process. Fourth, a comprehensive search was conducted across multiple electronic databases to identify potentially eligible studies. Finally, study selection, quality appraisal, and data extraction were independently performed by two reviewers, with disagreements resolved through discussion with a third reviewer.

### Limitations

This review has four key limitations. First, the possibility of a publication bias cannot be ignored because only Chinese or English articles were included in our SR, resulting in the exclusion of relevant studies published in other languages. Second, the included studies generally had small sample sizes, with a median of 50 participants, resulting in wide CIs and low precision. Third, the studies varied in terms of treatment duration (range: 4 weeks to 3.5 years) and melatonin administration time (at night, before bedtime, or 1 to 2 h before bedtime). To address this, we performed subgroup analyses. Fourth, for some subgroups with moderate to high heterogeneity (e.g., changes in cognitive functions from 13 to 25 weeks as measured by MMSE), meta-regression is recommended in future reviews to examine treatment effects while adjusting for differences across studies and identify study-level covariates that may explain heterogeneity [37]. Fifth, the inclusion of only Chinese and English studies in this review may limit the generalizability of the results. Finally, treatment adherence was not reported; this reduces the accuracy of the results.

### Implications for future studies and practice

Our findings highlight a need for large-scale studies. In addition, we strongly recommend conducting individual participant data meta-analyses or network meta-analyses to examine effect moderators more comprehensively in future studies. Future research should analyze the effects of melatonin across treatment durations, dosages, and patient characteristics. Longitudinal methodologically rigorous studies are required to confirm the effects of melatonin on cognitive function. On the basis of our findings, we recommend melatonin for adults with MCI. For optimal benefits, melatonin should be administered between 20:30 and 21:00 for 13–24 weeks.

Although melatonin is regarded as a supplement instead of a prescription medication, it is important to consider its safety, tolerability, and adverse effects when administering it. Tuft et al. reviewed primary studies that reported the maximum dosages of up to 1600 mg per day [14]. However, it is suggested to start with a lower dosage of 1–2 mg daily [38] and avoid exceeding 10 mg daily, as a higher dosage is associated with a higher rate of adverse effects [14].

## Conclusion

This SR supports the benefits of melatonin in adults with cognitive impairment, particularly those with MCI, when administered between 20:30 and 21:00 for 13–24 weeks. Melatonin appears to be relatively safe for individuals with cognitive impairment. However, individual participant data meta-analyses or network meta-analyses incorporating meta-regression are recommended to be conducted in future. In addition, further clinical trials with robust methods and large sample sizes are necessary to confirm this.

## Supplementary Information

Supplementary Material 1.

## Data Availability

No datasets were generated or analysed during the current study.

## Abbreviations

AD: Alzheimer’s disease
ADAS-Cog: Alzheimer’s Disease Assessment Scale–Cognitive Subscale
AE: Adverse effect
CI: Confidence interval
MCI: Mild cognitive impairment
MD: Mean difference
MMSE: Mini-Mental State Examination
PEDro: Physiotherapy Evidence Database
SR: Systematic review
