# Supplementary material for: Effect of melatonin on cognitive function in adults with cognitive impairment: a multi-dimensional meta-analysis of randomized trials
Source: Alzheimers Res Ther. 2025 Nov 3;17:238. doi: 10.1186/s13195-025-01881-w (PMC12581400; doi:10.1186/s13195-025-01881-w)
Supplement: Supplementary file 1 — Supplementary Material 1. [file 13195_2025_1881_MOESM1_ESM.docx]

**Supplementary Content**

**Supplementary S-Table 1. Search strategy for the seven databases.**

**Supplementary S-Table 2. PEDro Appraisal tool.**

**Supplementary S-Table 3.** List of excluded SRs.

**Supplementary S-Table 4. Quality of the included studies.**

**Supplementary S-Table 5. Sensitivity analyses of the included studies.**

**Supplementary S-Table 6. Supplementary meta-analysis results of the included studies.**

**Supplementary S-Table 7. Adverse events of the included studies.**

**Supplementary S-Table 8. PRISMA checklist 2020.**

**Supplementary S-Table 9. PRISMA abstract checklist 2020.**

**Supplementary S-Table 1.1: Search strategy for PubMed as 14 April 2025.**

| **Search** | **Query** |
| --- | --- |
| #1 | cognitive function.mp. or Cognition/ |
| #2 | melatonin.mp. or Melatonin/ |
| #3 | Randomized Controlled Trials as Topic/ or Adult/ or Male/ or Middle Aged/ or Female/ or Humans/ or randomi* control* trial*.mp. or Aged/ |
| #4 | Male/ or Female/ or Aged/ or Cognitive Dysfunction/ or Humans/ or Alzheimer Disease/ or alzheimer*.mp. or Dementia/ |
| #5 | Middle Aged/ or Male/ or Adult/ or Female/ or Humans/ or Aging/ or Aged/ or older adult*.mp. or "Aged, 80 and over"/ |
| #6 | 1 and 2 and 3 and 4 and 5 |

**Supplementary S-Table 1.2: Search strategy for Web of Science as at 15 April 2025.**

| **Search** | **Query** |
| --- | --- |
| #1 | TI=(melatonin*) OR AB=(melatonin*) OR TS=(melatonin*) OR TI=(N-cetyl-5-methoxytryptamine*) OR AB=(N-cetyl-5-methoxytryptamine*) OR TS=(N-cetyl-5-methoxytryptamine*) |
| #2 | TI=(cognitive function*) OR AB=(cognitive function*) OR TS=(cognitive function*) OR TI=(cognition*) OR AB=(cognition*) OR TS=(cognition*) OR TI=(cognitive*) OR AB=(cognitive*) OR TS=(cognitive*) |
| #3 | TI=(elder*) OR AB=(elder*) OR TS=(elder*) OR TI=(older adult*) OR AB=(older adult*) OR TS=(older adult*) OR TI=(age*) OR AB=(age*) OR TS=(age*) |
| #4 | TI=(dementia*) OR AB=(dementia*) OR TS=(dementia*) OR TI=(alzheimer*) OR AB=(alzehimer*) OR TS=(alzheimer*) |
| #5 | **TI=(randomi*) OR AB=(randomi*) OR TS=(randomi*)** |
| #6 | #1 AND #2 AND #3 AND #4 AND #5 |
| #7 | **TI=(review*) OR TI=(protocol)** |
| #8 | **#6 NOT #7** |

**Supplementary S-Table 1.3: Search strategy for EMBASE as at 16 April 2025.**

| **Search** | **Query** |
| --- | --- |
| #1 | melatonin*:ti,ab,kw OR 'n cetyl 5 methoxytryptamine*':ti,ab,kw |
| #2 | cognitive AND function*:ti,ab,kw OR 'cognition*':ti,ab,kw OR 'cognitive*':ti,ab,kw |
| #3 | elder*:ti,ab,kw OR 'older adult*':ti,ab,kw OR 'age*':ti,ab,kw |
| #4 | dementia*:ti,ab,kw OR 'alzheimer*':ti,ab,kw |
| #5 | randomi*:ti,ab,kw |
| #6 | #1 AND #2 AND #3 AND #4 AND #5 |
| #7 | review*:ti AND protocol*:ti |
| #8 | #6 NOT #7 |

**Supplementary S-Table 1.4: Search strategy for CINAHL as at 16 April 2025.**

| **Search** | **Query** |
| --- | --- |
| #1 | MW Melatonin* OR MW N-acetyl-5-methoxytryptamine* |
| #2 | MW Alzheimer* OR MW Dementia* |
| #3 | MW (elderly or aged or older or elder or geriatric or elderly people or old people or old people or senior) |
| #4 | MW (cognition or cognitive function or cognitive performance or cognitive abilities or cognitive ability) |
| #5 | AB randomi* |
| #6 | (AB randomi*) AND (S1 AND S2 AND S3 AND S4 AND S5) |

**Supplementary S-Table 1.5: Search strategy for SCOPUS as at 16 April 2025.**

| **Search** | **Query** |
| --- | --- |
| #1 | TITLE-ABS-KEY ( melatonin* OR n-acetyl-5-methoxytryptamine* ) |
| #2 | TITLE-ABS-KEY ( alzheimer* OR dementia* ) |
| #3 | TITLE-ABS-KEY ( cognitive AND function* OR cognition* OR cognitive* ) |
| #4 | TITLE-ABS-KEY ( elder* OR older AND adult* OR age* ) |
| #5 | TITLE-ABS-KEY ( randomi* ) |
| #6 | #1 AND #2 AND #3 AND #4 AND #5 |
| #7 | TITLE-ABS-KEY ( review* OR protocol ) |
| #8 | #6 NOT #7 |

**Supplementary S-Table 1.6: Search strategy for Cochrane as at 16 April 2025.**

| **Search** | **Query** |
| --- | --- |
| #1 | Melatonin* |
| #2 | Alzheimer* OR Dementia* |
| #3 | elderly* OR age* OR older adult* OR old people OR geriatric* |
| #4 | cognition* or cognitive function* or cognitive performance* or cognitive abilities or cognitive ability |
| #5 | randomi* |
| #6 | #1 and #2 and #3 and #4 and #5 |

**Supplementary file 1.7: Search strategy for CNKI as at 16 April 2025.**

| **Search** | **Query** |
| --- | --- |
| #1 | Title, Keyword and Abstract：認知功能障礙 |
| #2 | Title, Keyword and Abstract ：褪黑素 |
| #3 | #1 and #2 |

**Supplementary S-Table 2: PEDro Appraisal tool**

| Items | Content | Options |
| --- | --- | --- |
| 1 | Eligibility criteria were specified | Yes, No |
| 2 | Subjects were randomly allocated to groups (in a crossover study, subjects were randomly allocated an order in which treatments were received) | Yes, No |
| 3 | Allocation was concealed | Yes, No |
| 4 | The groups were similar at baseline regarding the most important prognostic indicators | Yes, No |
| 5 | There was blinding of all subjects | Yes, No |
| 6 | There was blinding of all therapists who administered the therapy | Yes, No |
| 7 | There was blinding of all assessors who measured at least one key outcome | Yes, No |
| 8 | Measures of at least one key outcome were obtained from more than 85% of the subjects initially allocated to groups | Yes, No |
| 9 | All subjects for whom outcome measures were available received the treatment or control condition as allocated or, where this was not the case, data for at least one key outcome was analysed by “intention to treat” | Yes, No |
| 10 | The results of between-group statistical comparisons are reported for at least one key outcome | Yes, No |
| 11 | The study provides both point measures and measures of variability for at least one key outcome | Yes, No |

Verhagen, A. P., de Vet H. C., de Bie, R. A., Kessels, A. G., Boers, M., Bouter, L. M., & Knipschild, P. G. (1998). The Delphi list: a criteria list for quality assessment of randomised clinical trials for conducting systematic reviews developed by Delphi consensus. *Journal of Clinical Epidemiology, 51*(12),1235-1241.

**Notes on administration of the PEDro scale:**

All criteria **Points are only awarded when a criterion is clearly satisfied**. If on a literal reading of the trial report it is possible that a criterion was not satisfied, a point should not be awarded for that criterion.

| Criteria | Description |
| --- | --- |
| 1 | This criterion is satisfied if the report describes the source of subjects and a list of criteria used to determine who was eligible to participate in the study |
| 2 | A study is considered to have used random allocation if the report states that allocation was random. The precise method of randomisation need not be specified. Procedures such as coin-tossing and dice-rolling should be considered random. Quasi-randomisation allocation procedures such as allocation by hospital record number or birth date, or alternation, do not satisfy this criterion. |
| 3 | *Concealed allocation* means that the person who determined if a subject was eligible for inclusion in the trial was unaware, when this decision was made, of which group the subject would be allocated to. A point is awarded for this criteria, even if it is not stated that allocation was concealed, when the report states that allocation was by sealed opaque envelopes or that allocation involved contacting the holder of the allocation schedule who was “off-site”. |
| 4 | At a minimum, in studies of therapeutic interventions, the report must describe at least one measure of the severity of the condition being treated and at least one (different) key outcome measure at baseline. The rater must be satisfied that the groups’ outcomes would not be expected to differ, on the basis of baseline differences in prognostic variables alone, by a clinically significant amount. This criterion is satisfied even if only baseline data of study completers are presented. |
| 4, 7-11 | *Key outcomes* are those outcomes which provide the primary measure of the effectiveness (or lack of effectiveness) of the therapy. In most studies, more than one variable is used as an outcome measure. |
| 5-7 | *Blinding* means the person in question (subject, therapist or assessor) did not know which group the subject had been allocated to. In addition, subjects and therapists are only considered to be “blind” if it could be expected that they would have been unable to distinguish between the treatments applied to different groups. In trials in which key outcomes are self-reported (eg, visual analogue scale, pain diary), the assessor is considered to be blind if the subject was blind. |
| 8 | This criterion is only satisfied if the report explicitly states *both* the number of subjects initially allocated to groups *and* the number of subjects from whom key outcome measures were obtained. In trials in which outcomes are measured at several points in time, a key outcome must have been measured in more than 85% of subjects at one of those points in time. |
| 9 | An *intention to treat* analysis means that, where subjects did not receive treatment (or the control condition) as allocated, and where measures of outcomes were available, the analysis was performed as if subjects received the treatment (or control condition) they were allocated to. This criterion is satisfied, even if there is no mention of analysis by intention to treat, if the report explicitly states that all subjects received treatment or control conditions as allocated. |
| 10 | *A between-group* statistical comparison involves statistical comparison of one group with another. Depending on the design of the study, this may involve comparison of two or more treatments, or comparison of treatment with a control condition. The analysis may be a simple comparison of outcomes measured after the treatment was administered, or a comparison of the change in one group with the change in another (when a factorial analysis of variance has been used to analyse the data, the latter is often reported as a group  time interaction). The comparison may be in the form hypothesis testing (which provides a “p” value, describing the probability that the groups differed only by chance) or in the form of an estimate (for example, the mean or median difference, or a difference in proportions, or number needed to treat, or a relative risk or hazard ratio) and its confidence interval. |
| 11 | A *point measure* is a measure of the size of the treatment effect. The treatment effect may be described as a difference in group outcomes, or as the outcome in (each of) all groups. *Measures of variability* include standard deviations, standard errors, confidence intervals, interquartile ranges (or other quantile ranges), and ranges. Point measures and/or measures of variability may be provided graphically (for example, SDs may be given as error bars in a Figure) as long as it is clear what is being graphed (for example, as long as it is clear whether error bars represent SDs or SEs). Where outcomes are categorical, this criterion is considered to have been met if the number of subjects in each category is given for each group. |

Supplementary **S-Table** 3: List of excluded studies (N = 65).

| No. | Reference | Title | Reason for exclusion |
| --- | --- | --- | --- |
| 1 | Lawlor, P. G., McNamara-Kilian, M. T., MacDonald, A. R., Momoli, F., Tierney, S., Lacaze-Masmonteil, N., Dasgupta, M., Agar, M., Pereira, J. L., Currow, D. C., & Bush, S. H. (2020). | Melatonin to prevent delirium in patients with advanced cancer: a double blind, parallel, randomized, controlled, feasibility trial. *BMC palliative care*, *19*(1), 163. https://doi.org/10.1186/s12904-020-00669-z | Wrong outcome |
| 2 | de Jonghe, A., van Munster, B. C., Goslings, J. C., Kloen, P., van Rees, C., Wolvius, R., van Velde, R., Levi, M., de Haan, R. J., de Rooij, S. E., & Amsterdam Delirium Study Group (2014). | Effect of melatonin on incidence of delirium among patients with hip fracture: a multicentre, double-blind randomized controlled trial. *CMAJ : Canadian Medical Association journal*, *186*(14), E547–E556. https://doi.org/10.1503/cmaj.140495 | Wrong outcome |
| 3 | Cremascoli, R., Sparasci, D., Giusti, G., Cattaldo, S., Prina, E., Roveta, F., Bruno, F., Ghezzi, C., Cerri, S., Picascia, M., Bernini, S., Sinforiani, E., Terzaghi, M., Priano, L., Mauro, A., & Manni, R. (2022). | Effects of Circadian Phase Tailored Light Therapy on Sleep, Mood, and Cognition in Alzheimer's Disease: Preliminary Findings in a Pivotal Study. *Frontiers in physiology*, *12*, 755322. https://doi.org/10.3389/fphys.2021.755322 | Wrong intervention |
| 4 | Cazzola, R., Rondanelli, M., Faliva, M., & Cestaro, B. (2012). | Effects of DHA-phospholipids, melatonin and tryptophan supplementation on erythrocyte membrane physico-chemical properties in elderly patients suffering from mild cognitive impairment. *Experimental gerontology*, *47*(12), 974–978. https://doi.org/10.1016/j.exger.2012.09.004 | Mixed intervention |
| 5 | Otmani, S., Demazières, A., Staner, C., Jacob, N., Nir, T., Zisapel, N., & Staner, L. (2008). | Effects of prolonged-release melatonin, zolpidem, and their combination on psychomotor functions, memory recall, and driving skills in healthy middle aged and elderly volunteers. *Human psychopharmacology*, *23*(8), 693–705. https://doi.org/10.1002/hup.980 | Wrong outcome |
| 6 | Fan, Y., Yuan, L., Ji, M., Yang, J., & Gao, D. (2017). | The effect of melatonin on early postoperative cognitive decline in elderly patients undergoing hip arthroplasty: A randomized controlled trial. *Journal of clinical anesthesia*, *39*, 77–81. https://doi.org/10.1016/j.jclinane.2017.03.023 | Wrong subjects |
| 7 | Ford, A. H., Flicker, L., Passage, J., Wibrow, B., Anstey, M., Edwards, M., & Almeida, O. P. (2016). | The Healthy Heart-Mind trial: melatonin for prevention of delirium following cardiac surgery: study protocol for a randomized controlled trial. *Trials*, *17*, 55. https://doi.org/10.1186/s13063-016-1163-1 | Protocol |
| 8 | Martin, J. L., Marler, M. R., Harker, J. O., Josephson, K. R., Alessi, C. A. (2007). | A Multicomponent Nonpharmacological Intervention Improves Activity Rhythms Among Nursing Home Residents With Disrupted Sleep/Wake Patterns, The Journals of Gerontology: Series A, 62(1), 67–72, <https://doi.org/10.1093/gerona/62.1.67> | Wrong intervention |
| 9 | Cardinali, D. P., Furio, A. M., & Brusco, L. I. (2010). | Clinical aspects of melatonin intervention in Alzheimer's disease progression. *Current neuropharmacology*, *8*(3), 218–227. https://doi.org/10.2174/157015910792246209 | Review |
| 10 | Duffy, J. F., Wang, W., Ronda, J. M., & Czeisler, C. A. (2022). | High dose melatonin increases sleep duration during nighttime and daytime sleep episodes in older adults. *Journal of pineal research*, *73*(1), e12801. https://doi.org/10.1111/jpi.12801 | Wrong intervention |
| 11 | Siddiqi, N., Harrison, J. K., Clegg, A., Teale, E. A., Young, J., Taylor, J., Simpkins, S. A. (2016). | Interventions for preventing delirium in hospitalised non-ICU patients. *Cochrane Database of Systematic Reviews,* 3. Art. No.: CD005563. doi: 10.1002/14651858.CD005563.pub3. | Review |
| 12 | Forbes, D., Culum, I., Lischka, A. R., Morgan, D. G., Peacock, S., Forbes, J., Forbes, S. (2009). | Light therapy for managing cognitive, sleep, functional, behavioural, or psychiatric disturbances in dementia.  *Cochrane Database of Systematic Reviews,* 4. Art. No.: CD003946. doi: 10.1002/14651858.CD003946.pub3. | Review |
| 13 | Alagiakrishnan, K. (2016) | Melatonin based therapies for delirium and dementia. Discovery Medicine, 21(117), 363-371. | Wrong outcome |
| 14 | Jaiswal, S. J., Kang, D. Y., Wineinger, N. E., Owens, R. L. (2020). | Objectively measured sleep fragmentation is associated with incident delirium in older hospitalized patients: Analysis of data collected from an randomized controlled trial. Journal of Sleep Research, 30, e13205. Doi: 10.1111/jsr.13205 | Wrong outcome |
| 15 | Katsuta, N., Takahashi, K., Kurosawa, Y., Yoshikawa, A., Takeshita, Y., Uchida, Y., Yasuda, S., Kakiuchi, C., Ito, M., Kato, T. (2023). | Safety and real-world efficacy of lemborexant in the treatment of comorbid insomnia. *Sleep Medicine, X*, 5. <https://doi.org/10.1016/j.sleepx.2023.100070> | Wrong intervention |
| 16 | Schneider, L. S., Laudon, M., Nir, T., Caceres, J., Ianniciello, G., Capulli, M., & Zisapel, N. (2022). | A Polymorphism Cluster at the 2q12 locus May Predict Response to Piromelatine in Patients with Mild Alzheimer's Disease. *The journal of prevention of Alzheimer's disease*, *9*(2), 247–254. https://doi.org/10.14283/jpad.2021.61 | Wrong intervention |
| 17 | Glass, O. M., Hermida, A. P., Hershenberg, R., & Schwartz, A. C. (2020). | Considerations and Current Trends in the Management of the Geriatric Patient on a Consultation-Liaison Service. *Current psychiatry reports*, *22*(5), 21. https://doi.org/10.1007/s11920-020-01147-2 | Review |
| 18 | Rondanelli, M., Opizzi, A., Faliva, M., Mozzoni, M., Antoniello, N., Cazzola, R., Savarè, R., Cerutti, R., Grossi, E., & Cestaro, B. (2012). | Effects of a diet integration with an oily emulsion of DHA-phospholipids containing melatonin and tryptophan in elderly patients suffering from mild cognitive impairment. *Nutritional neuroscience*, *15*(2), 46–54. https://doi.org/10.1179/1476830511Y.0000000032 | Mixed intervention |
| 19 | Schneider, L., Laudon, M., Caceres, T. N. J., Ianniciello, G., Capulli, M., & Zisapel, N. (2021). | A polymorphism cluster at the 2Q12 locus may predict response to piromelatine in patients with mild Alzheimer’s disease. *The Journal of Prevention of Alzheimer’s Disease, 8*(1), OC10. | Duplicate with #16 |
| 20 | Vecchierini, M. F., Kilic-Huck, U., Quera-Salva, M. A., & Members of the MEL consensus group of the SFRMS (2021). | Melatonin (MEL) and its use in neurological diseases and insomnia: Recommendations of the French Medical and Research Sleep Society (SFRMS). *Revue neurologique*, *177*(3), 245–259. https://doi.org/10.1016/j.neurol.2020.06.009 | Review |
| 21 | Morales-Delgado, R., Cámara-Lemarroy, C. R., Salinas-Martínez, R., Gámez-Treviño, D., Arredondo-Jaime, A., Hernández-Maldonado, E., & Guajardo-Álvarez, G. (2018). | A randomized placebo-controlled trial evaluating the effect of melatonin on sleep quality in patients with mild-moderate dementia. *European geriatric medicine*, *9*(4), 449–454. https://doi.org/10.1007/s41999-018-0068-9 | Wrong outcome |
| 22 | Wilt, T. J., MacDonald, R., Brasure, M., Olson, C. M., Carlyle, M., Fuchs, E., Khawaja, I. S., Diem, S., Koffel, E., Ouellette, J., Butler, M., Kane, R. L. (2016). | [Pharmacologic Treatment of Insomnia Disorder: An Evidence Report for a Clinical Practice Guideline by the American College of Physicians](https://www.acpjournals.org/doi/abs/10.7326/M15-1781). *Annals of Internal Medicine, 165*, 103-112.  doi:[10.7326/M15-1781](https://doi.org/10.7326/M15-1781) | Review |
| 23 | Obayashi, K., Saeki, K., Iwamoto, J., Tone, N., Tanaka, K., Kataoka, H., Morikawa, M., & Kurumatani, N. (2015). | Physiological Levels of Melatonin Relate to Cognitive Function and Depressive Symptoms: The HEIJO-KYO Cohort. *The Journal of clinical endocrinology and metabolism*, *100*(8), 3090–3096. https://doi.org/10.1210/jc.2015-1859 | Wrong outcome |
| 24 | Hu, K., Riemersma-van der Lek, R. F., Patxot, M., Li, P., Shea, S. A., Scheer, F. A., & Van Someren, E. J. (2016). | Progression of Dementia Assessed by Temporal Correlations of Physical Activity: Results From a 3.5-Year, Longitudinal Randomized Controlled Trial. *Scientific reports*, *6*, 27742. https://doi.org/10.1038/srep27742 | Wrong intervention |
| 25 | Baandrup, L., Lindschou, J., Winkel, P., Gluud, C., & Glenthoj, B. Y. (2016). | Prolonged-release melatonin versus placebo for benzodiazepine discontinuation in patients with schizophrenia or bipolar disorder: A randomised, placebo-controlled, blinded trial. *The world journal of biological psychiatry : the official journal of the World Federation of Societies of Biological Psychiatry*, *17*(7), 514–524. https://doi.org/10.3109/15622975.2015.1048725 | Protocol |
| 26 | Schenck, C. H., Montplaisir, J. Y., Frauscher, B., Hogl, B., Gagnon, J. F., Postuma, R., Sonka, K., Jennum, P., Partinen, M., Arnulf, I., Cochen de Cock, V., Dauvilliers, Y., Luppi, P. H., Heidbreder, A., Mayer, G., Sixel-Döring, F., Trenkwalder, C., Unger, M., Young, P., Wing, Y. K., … Oertel, W. (2013). | Rapid eye movement sleep behavior disorder: devising controlled active treatment studies for symptomatic and neuroprotective therapy--a consensus statement from the International Rapid Eye Movement Sleep Behavior Disorder Study Group. *Sleep medicine*, *14*(8), 795–806. https://doi.org/10.1016/j.sleep.2013.02.016 | Review |
| 27 | Bliwise D. L. (2004). | Sleep disorders in Alzheimer's disease and other dementias. *Clinical cornerstone*, *6 Suppl 1A*, S16–S28. https://doi.org/10.1016/s1098-3597(04)90014-2 | Wrong study design |
| 28 | Cardinali, D. P., Furio, A. M., & Brusco, L. I. (2011). | The use of chronobiotics in the resynchronization of the sleep/wake cycle. Therapeutical application in the early phases of Alzheimer's disease. *Recent patents on endocrine, metabolic & immune drug discovery*, *5*(2), 80–90. https://doi.org/10.2174/187221411799015354 | Wrong study design |
| 29 | de Jonghe, A.,van Munster, B. C., Goslings, J. C., Kloen, P., van Rees, C., Wolvius, R., van Velde, R., Levi, M. M., & de Hann, R. J. (2013). | A randomized, double-blind, controlled trial of melatonin versus placebo in delirium. *European Geriatric Medicine, 4,* S142-S216, 521.  http://dx.doi.org/10.1016/j.eurger.2013.07.586 | Abstract |
| 30 | Laborie, S. (2010). | Effect of bright light and melatonin on cognitive and non-cognitive function. [Les Cahiers de l'année gérontologique,](https://data.bnf.fr/41340950/les_cahiers_de_l_annee_gerontologique__en_ligne_/) 2, 194-198. doi:10.1007/s12612-010-0161-2 | Not in English |
| 31 | Van Someren, E. J. W., Riemersma, R. F., Swaab, D. F. (2016). | Effects of light on the sleeping-waking pattern in the elderly and in dementia. *Tijdschrift voor psychiatrie, 47*, 29-38. | Not in English |
| 32 | Menczel Schrire, Z., Phillips, C. L., Duffy, S. L., Marshall, N. S., Mowszowski, L., La Monica, H. M., Gordon, C. J., Chapman, J. L., Saini, B., Lewis, S. J. G., Naismith, S. L., Grunstein, R. R., & Hoyos, C. M. (2021). | Feasibility of 3-month melatonin supplementation for brain oxidative stress and sleep in mild cognitive impairment: protocol for a randomised, placebo-controlled study. *BMJ open*, *11*(2), e041500. https://doi.org/10.1136/bmjopen-2020-041500 | Protocol |
| 33 | Miyamoto, M., & Miyamoto, T. (2019). | Preparation and future direction of clinical trials for idiopathic REM sleep behavior disorder. *Clinical Neurology, 59*, S35. | Abstract |
| 34 | Hatta, K. (2015). | Preventive effects of ramelteon on delirium in patients with mild cognitive impairment or dementia. International *Psychogeriatrics, 27*(1), S6-S44. doi:10.1017/S1041610215002148 | Abstract |
| 35 | Sarraf, N., Badri, T., Keshvari, N., Ghassab-sahebkar, A., Qobadighadikolaei, R., & Abbasinazari, M. (2020). | Comparison of the efficacy and safety of melatonin and memantine in the alleviation of cognitive impairments induced by electroconvulsive therapy: a randomized controlled trial. Journal of Clinical Neuroscience, 74, 146-150. doi: [10.1016/j.jocn.2020.02.011](https://doi.org/10.1016/j.jocn.2020.02.011) | Wrong intervention |
| 36 | Slotten, H. A., & Krekling, S. (1997). | Does melatonin have an effect on cognitive performance? *Psychoneuroendocrinology, 21*, 9, 673-680. | Wrong outcome |
| 37 | Kamoun, A., Hammouda, O., Yahia, A., Dhari, O., Ksentini, H., Driss, T., Souissi, N., & Elleuch, M. H. (2019). | Effects of Melatonin Ingestion Before Nocturnal Sleep on Postural Balance and Subjective Sleep Quality in Older Adults. Journal of Aging and Physical Activity, 27(3), 316-324. doi: 10.1123/japa.2018-0107 | Wrong outcome |
| 38 | Kurowska, A., Bodys-Cupak, I., Staszkiewicz, M., Szklarczyk, J., Zalewska-Puchała, J., Kliś-Kalinowska, A., Makara-Studzińska, M., & Majda, A. (2020). | Interleukin-6 and Melatonin as Predictors of Cognitive, Emotional and Functional Ageing of Older People. *International journal of environmental research and public health*, *17*(10), 3623. | Non-RCT |
| 39 | Jean-Louis, G., von Gizycki, H., & Zizi, F. (1998). | Melatonin effects on sleep, mood, and cognition in elderly with mild cognitive impairment. *Journal of pineal research*, *25*(3), 177–183. https://doi.org/10.1111/j.1600-079x.1998.tb00557.x | Wrong outcome |
| 40 | Kim, S. J., Lee, S. H., Suh, I. B., Jang, J. W., Jhoo, J. H., & Lee, J. H. (2021). | Positive effect of timed blue-enriched white light on sleep and cognition in patients with mild and moderate Alzheimer's disease. *Scientific reports*, *11*(1), 10174. https://doi.org/10.1038/s41598-021-89521-9 | Wrong intervention |
| 41 | Davis, G. R., Etheredge, C. E., Marcus, L., & Bellar, D. (2014). | Prolonged sleep deprivation and continuous exercise: effects on melatonin, tympanic temperature, and cognitive function. *BioMed research international*, *2014*, 781863. https://doi.org/10.1155/2014/781863 | Wrong outcome |
| 42 | Polvat, T., Prasertporn, T., Na Nakorn, P., Pannengpetch, S., Suwanjang, W., Panmanee, J., Ngampramuan, S., Cornish, J. L., & Chetsawang, B. (2023). | Proteomic Analysis Reveals the Neurotoxic Effects of Chronic Methamphetamine Self-Administration-Induced Cognitive Impairments and the Role of Melatonin-Enhanced Restorative Process during Methamphetamine Withdrawal. *Journal of proteome research*, *22*(10), 3348–3359. https://doi.org/10.1021/acs.jproteome.3c00502 | Animal study |
| 43 | Andrade C. (2022). | Reconsideration of the Benefits of Pharmacological Interventions for the Attenuation of the Cognitive Adverse Effects of Electroconvulsive Therapy. *The Journal of clinical psychiatry*, *83*(5), 22f14668. https://doi.org/10.4088/JCP.22f14668 | Animal study |
| 44 | Naguib, M., & Samarkandi, A. H. (2000). | The comparative dose-response effects of melatonin and midazolam for premedication of adult patients: a double-blinded, placebo-controlled study. *Anesthesia and analgesia*, *91*(2), 473–479. https://doi.org/10.1097/00000539-200008000-00046 | Wrong outcome |
| 45 | Kwon, K. J., Lee, E. J., Kim, M. K., Jeon, S. J., Choi, Y. Y., Shin, C. Y., & Han, S. H. (2015). | The potential role of melatonin on sleep deprivation-induced cognitive impairments: implication of FMRP on cognitive function. *Neuroscience*, *301*, 403–414. https://doi.org/10.1016/j.neuroscience.2015.05.079 | Wrong study design |
| 46 | Rutten, S., Vriend, C., Smit, J. H., Berendse, H. W., Hoogendoorn, A. W., van den Heuvel, O. A., & van der Werf, Y. D. (2016). | A double-blind randomized controlled trial to assess the effect of bright light therapy on depression in patients with Parkinson's disease. *BMC psychiatry*, *16*(1), 355. https://doi.org/10.1186/s12888-016-1050-z | Wrong intervention |
| 47 | Wilhelmsen-Langeland, A., Saxvig, I. W., Pallesen, S., Nordhus, I. H., Vedaa, Ø., Lundervold, A. J., & Bjorvatn, B. (2013). | A randomized controlled trial with bright light and melatonin for the treatment of delayed sleep phase disorder: effects on subjective and objective sleepiness and cognitive function. *Journal of biological rhythms*, *28*(5), 306–321. https://doi.org/10.1177/0748730413500126 | Wrong intervention |
| 48 | Devore, E. E., Harrison, S. L., Stone, K. L., Holton, K. F., Barrett-Connor, E., Ancoli-Israel, S., Yaffe, K., Ensrud, K., Cawthon, P. M., Redline, S., Orwoll, E., Schernhammer, E. S., & Osteoporotic Fractures in Men (MrOS) Study Research Group (2016). | Association of urinary melatonin levels and aging-related outcomes in older men. *Sleep medicine*, *23*, 73–80. https://doi.org/10.1016/j.sleep.2016.07.006 | Wrong outcome |
| 49 | Palmer, A. C. S., Zortea, M., Souza, A., Santos, V., Biazús, J. V., Torres, I. L. S., Fregni, F., & Caumo, W. (2020). | Clinical impact of melatonin on breast cancer patients undergoing chemotherapy; effects on cognition, sleep and depressive symptoms: A randomized, double-blind, placebo-controlled trial. *PloS one*, *15*(4), e0231379. https://doi.org/10.1371/journal.pone.0231379 | Wrong subject |
| 50 | Reyt, .M, Deantoni, M., Baillet, M., Lesoinne, A., Laloux, S., Lambot, E., Demeuse, J., Calaprice, C., LeGoff, C., Collette, F., Vandewalle, G., Maquet, P., Muto, V., Hammad, G., & Christina, S. (2022). | Daytime rest: Association with 24-h rest-activity cycles, circadian timing and cognition in older adults. Journal of pineal research, 2022-10, Vol.73 (3), p.e12820-n/a | Wrong study design |
| 51 | Dollins, A. B., Lynch, H. J., Wurtman, R. J., Deng, M. H., Kischka, K. U., Gleason, R. E., & Lieberman, H. R. (1993). | Effect of pharmacological daytime doses of melatonin on human mood and performance. *Psychopharmacology*, *112*(4), 490–496. https://doi.org/10.1007/BF02244899 | Wrong outcome |
| 52 | Scheuer, C., Pommergaard, H. C., Rosenberg, J., & Gögenur, I. (2016). | Effect of topical application of melatonin cream 12.5% on cognitive parameters: A randomized, placebo-controlled, double-blind crossover study in healthy volunteers. *Journal of Dermatological Treatment*, *27*(6), 488–494. https://doi.org/10.3109/09546634.2016.1161154 | Wrong intervention |
| 53 | Hadoush, H., Lababneh, T., Banihani, S. A., Al-Jarrah, M., & Jamous, M. (2020). | Melatonin and dopamine serum level associations with motor, cognitive, and sleep dysfunctions in patients with Parkinson's disease: A cross-sectional research study. *NeuroRehabilitation*, *46*(4), 539–549. https://doi.org/10.3233/NRE-203075 | Wrong study design |
| 54 | Burkhalter, H., Wirz-Justice, A., Denhaerynck, K., Fehr, T., Steiger, J., Venzin, R. M., Cajochen, C., Weaver, T. E., & De Geest, S. (2015). | The effect of bright light therapy on sleep and circadian rhythms in renal transplant recipients: a pilot randomized, multicentre wait-list controlled trial. *Transplant international : official journal of the European Society for Organ Transplantation*, *28*(1), 59–70. https://doi.org/10.1111/tri.12443 | Wrong intervention |
| 55 | Lockley S. W. (2005). | Timed melatonin treatment for delayed sleep phase syndrome: the importance of knowing circadian phase. *Sleep*, *28*(10), 1214–1216. https://doi.org/10.1093/sleep/28.10.1214 | Wrong outcome |
| 56 | Garzón, C., Guerrero, J. M., Aramburu, O., & Guzmán, T. (2009). | Effect of melatonin administration on sleep, behavioral disorders and hypnotic drug discontinuation in the elderly: a randomized, double-blind, placebo-controlled study. *Aging clinical and experimental research*, *21*(1), 38–42. https://doi.org/10.1007/BF03324897 | Wrong intervention |
| 57 | Wade, A., Ford, I., Craw, G., McMahon, A., Nir, T., Laudon, M., & Zisapel, N. (2007). | Efficacy of prolonged release melatonin in insomnia patients aged 55–80 years: quality of sleep and next-day alertness outcomes. Current Medical Research and Opinion, 23(10), 2597-2605. doi:10.1185/030079907X233098 | Wrong outcome |
| 58 | Al-Aama, T., Brymer, C., Gutmanis, I., Woolmore-Goodwin, S. M., Esbaugh, J., & Dasgupta, M. (2011). | Melatonin decreases delirium in elderly patients: a randomized, placebo-controlled trial. *International journal of geriatric psychiatry*, *26*(7), 687–694. https://doi.org/10.1002/gps.2582 | Wrong outcome |
| 59 | Gehrman, P. R., Connor, D. J., Martin, J. L., Shochat, T., Corey-Bloom, J., & Ancoli-Israel, S. (2009). | Melatonin fails to improve sleep or agitation in double-blind randomized placebo-controlled trial of institutionalized patients with Alzheimer disease. *The American journal of geriatric psychiatry : official journal of the American Association for Geriatric Psychiatry*, *17*(2), 166–169. https://doi.org/10.1097/JGP.0b013e318187de18 | Wrong outcome |
| 60 | Mahlberg, R., & Walther, S. (2007). | Actigraphy in agitated patients with dementia. Monitoring treatment outcomes. *Zeitschrift fur Gerontologie und Geriatrie*, *40*(3), 178–184. https://doi.org/10.1007/s00391-007-0420-z | Wrong subject |
| 61 | Dowling, G. A., Burr, R. L., Van Someren, E. J., Hubbard, E. M., Luxenberg, J. S., Mastick, J., & Cooper, B. A. (2008). | Melatonin and bright-light treatment for rest-activity disruption in institutionalized patients with Alzheimer's disease. *Journal of the American Geriatrics Society*, *56*(2), 239–246. https://doi.org/10.1111/j.1532-5415.2007.01543.x | Wrong intervention |
| 62 | Gehrman, P. R., Connor, D. J., Martin, J. L., Shochat, T., Corey-Bloom, J., & Ancoli-Israel, S. (2009). | Melatonin fails to improve sleep or agitation in double-blind randomized placebo-controlled trial of institutionalized patients with Alzheimer disease. *The American journal of geriatric psychiatry : official journal of the American Association for Geriatric Psychiatry*, *17*(2), 166–169. https://doi.org/10.1097/JGP.0b013e318187de18 | Wrong outcome |
| 63 | Furio, A. M., Brusco, L. I., & Cardinali, D. P. (2007). | Possible therapeutic value of melatonin in mild cognitive impairment: a retrospective study. *Journal of pineal research*, *43*(4), 404–409. https://doi.org/10.1111/j.1600-079X.2007.00491.x | Wrong study design |
| 64 | Cardinali, D. P., Vigo, D. E., Olivar, N., Vidal, M. F., Furio, A. M., & Brusco, L. I. (2012). | Therapeutic application of melatonin in mild cognitive impairment. *American journal of neurodegenerative disease*, *1*(3), 280–291. | Wrong study design |
| 65 | Sultan S. S. (2010). | Assessment of role of perioperative melatonin in prevention and treatment of postoperative delirium after hip arthroplasty under spinal anesthesia in the elderly. *Saudi journal of anaesthesia*, *4*(3), 169–173. https://doi.org/10.4103/1658-354X.71132 | Wrong subject |
| 66 | Peck, J. S., LeGoff, D. B., Ahmed, I., & Goebert, D. (2004). | Cognitive effects of exogenous melatonin administration in elderly persons: a pilot study. *The American journal of geriatric psychiatry : official journal of the American Association for Geriatric Psychiatry*, *12*(4), 432–436. https://doi.org/10.1176/appi.ajgp.12.4.432 | Wrong subject |
| 67 | Serfaty, M., Kennell-Webb, S., Warner, J., Blizard, R., & Raven, P. (2002). | Double blind randomized placebo controlled trial of low dose melatonin for sleep disorders in dementia. *International Journal of Geriatric Psychiatric, 17*, 1120-1127. | No data on MMSE, author(s) contact but no reply. |
| 68 | Tavares, C., Memória, C. M., da Costa, L. G. V., Quintão, V. C., Antunes, A. A., Teodoro, D., & Carmona, M. J. C. (2024). | Effect of melatonin on postoperative cognitive function in elderly patients submitted to transurethral resection of the prostate under spinal anesthesia. *Clinics (Sao Paulo, Brazil)*, *80*, 100562. https://doi.org/10.1016/j.clinsp.2024.100562 | Wrong subject |

Supplementary **S-Table** 4: **Quality of the included studies (n = 8).**

| **No.** | **Authors** | **Year** | **1** | **2** | **3** | **4** | **5** | **6** | **7** | **8** | **9** | **10** | **11** | **Total** | **Comment** |
| --- | --- | --- | --- | --- | --- | --- | --- | --- | --- | --- | --- | --- | --- | --- | --- |
| 1 | Asayama et al. | 2003 | Yes | Yes | No | No | Yes | Yes | No | Yes | No | Yes | Yes | 6 | Good |
| 2 | Gao et al. | 2009 | Yes | Yes | No | Yes | No | Yes | No | Yes | No | Yes | Yes | 5 | Fair |
| 3 | Menczel Schrire et al. | 2024 | Yes | Yes | Yes | No | Yes | Yes | No | Yes | Yes | No | Yes | 8 | Good |
| 4 | Morales-Delgado et al. | 2008 | Yes | Yes | Yes | Yes | Yes | Yes | No | No | No | Yes | Yes | 8 | Good |
| 5 | Riemersma-van der Lek et al. | 2008 | Yes | Yes | Yes | Yes | Yes | Yes | No | Yes | Yes | Yes | Yes | 10 | Excellent |
| 6 | Singer et al. | 2003 | Yes | Yes | Yes | Yes | Yes | Yes | No | Yes | No | Yes | Yes | 8 | Good |
| 7 | Wade et al. | 2014 | Yes | Yes | Yes | Yes | Yes | Yes | No | No | No | Yes | Yes | 8 | Good |
| 8 | Xu et al. | 2002 | No | Yes | Yes | No | Yes | Yes | No | Yes | No | No | No | 5 | Fair |

**Answer "Yes" or "No" for the following questions:**

1. Eligibility criteria were specified

2. Subjects were randomly allocated to groups (in a crossover study, subjects were randomly allocated an order in which treatments were received)

3. Allocation was concealed

4. The groups were similar at baseline regarding the most important prognostic indicators

5. There was blinding of all subjects

6. There was blinding of all therapists who administered the therapy

7. There was blinding of all assessors who measured at least one key outcome

8. Measures of at least one key outcome were obtained from more than 85% of the subjects initially allocated to groups

9. All subjects for whom outcome measures were available received the treatment or control condition as allocated or, where this was not the case, data for at least one key outcome was analysed by “intention to treat”

10. The results of between-group statistical comparisons are reported for at least one key outcome

11. The study provides both point measures and measures of variability for at least one key outcome

**Results:**

<4 = poor

4-5 = fair

6-8 = good

9-10 = excellent

**Supplementary S-Table 5. Sensitivity analyses of change in cognitive functions in different dosage (MMSE).**

| **Author, year** | **I^2^** | **P-value** | **Meta-analysis Results** |
| --- | --- | --- | --- |
|  |  |  |  |
| *Original* | 87 | <0.00001 | 1.61(0.90, 2.32) |
| Singer (10mg, 8w) | 76 | <0.00001 | 2.31(1.49, 3.14) |
| Morales-Delgado (5mg, 8w) | 93 | <0.00001 | 1.77(1.02, 2.52) |
| Xu (0.15mg/kg, 6mo) | 0 | 0.53 | -0.40(-1.62, 0.83) |

**Remarks**: include fair results. The row with the author’s name and year indicated the result by removal of the author’s study.

**Supplementary S-Table 6. Adverse events of the included studies (n = 8).**

| **Author, Year, Country** | **Details of the adverse events** | **Authors’ conclusion** |
| --- | --- | --- |
| Asayama et al., (2003), Japan | N/A | N/A |
| Gao et al., (2009), China | No adverse events | N/A |
| Menczel Schrire (2024) | **Placebo:** head discomfort, faces discoloured, herpes zoster, tick removal, fatigue, fall, orbital infection, COVID-19, back pain, vertigo positional, musculoskeletal injury, sinusitis, sinus operational.  Melatonin: peripheral swelling, Raynaud’s phenomenon, atthralgia, diarrhoea infection, muscle spasms, fatigue, abnormal dreams, poor quality sleep, somnolence, limb fracture, fall, blastocystis infection, middle insomnia, COVID-19, constipation, giardiasis, sepsis. | No differences in adverse events between groups. |
| Morales-delgado et al., (2008), Mexico | No adverse events | N/A |
| Riemersma-van der Lek et al., (2008), Netherlands | Drowsiness, irritability, headache, eye complaints, dizziness, feebleness | Melatonin reduced the ratings on constipation. No severe adverse events were reported by the patients’ physicians. In the only case reported by others, the daughter of a 90-year old participant diagnosed with probable Lewybody dementia suspected her mother’s increase in restlessness and falls to be related to the treatment and requested discontinuation. This patient had been assigned to the double placebo group. |
| Singer et al., (2003), USA | **Placebo**: Abnormal behavior, ache/pain, falls, fatigue, gastrointestinal distress, infection, respiratory/pulmonary symptom, skin/subcutaneous tissue, urinary symptoms.  **ML 2.5SR**: Abnormal behavior, ache/pain, falls, gastrointestinal distress, infection, respiratory/ pulmonary symptom, skin/subcutaneous tissue, urinary symptoms.  **ML 10**: Abnormal behavior, ache/pain, falls, gastrointestinal distress, respiratory/pulmonary symptom, skin/subcutaneous tissue, urinary symptoms. | There were no significant differences in the number or seriousness of adverse events between the placebo and melatonin groups. |
| Wade et al., (2014), USA | **Placebo (N = 23)**: Abdominal discomfort. Diarrhea, Nausea, Vomiting, Fatigue, Blood creatinine increased, Blood glucose increased, Back pain, Agitation, Cognitive disorder, Insomnia, Urinary tract infection, Nasopharyngitis, Upper respiratory tract infection, Drug-related AEs, Fatigue, Headache  **Melatonin (N = 32)**: Angina pectoris, Abdominal discomfort. Diarrhea, Vomiting, Fatigue, Blood glucose increased, Decreased appetite, Back pain, Abnormal dreams, Agitation, Cognitive disorder, Insomnia, Urinary tract infection, Cough, Nasopharyngitis, Upper respiratory tract infection, Drug-related AEs, Fatigue, Fall, Thyroid neoplasm, Abnormal dreams, Burning feet syndrome, Somnolence, Delusion Restlessness | Prolonged-released melatonin was well tolerated, with an adverse event profile similar to that of placebo. |
| Xu et al., (2002), China | N/A | N/A |

**Supplementary S-Table 7**

With regards to two included studies (Gao et al., 2009 Riemersma-van et al. 2008) have different time point of measuring the outcomes, we conducted a supplementary meta-analysis on the different measurement points aimed to provide a more concrete and comprehensive information for the scholar to understand the effectiveness of melatonin on cognitive functions, at different dosages, different types of cognitive impairment, and different levels of dementia.

**Supplementary S-Table 7.1 (Data of Figure 2). Change in cognitive functions (MMSE).**

| **Author, year** | **I^2^** | **P-value** | **Meta-analysis Results** |
| --- | --- | --- | --- |
| Original | 53 | <0.0001 | 1.08(0.56, 1.60) |
| *MMSE* |  |  |  |
| **Gao (2.9mg, 12w)** | | | |
| Riemersma-van (2.5mg, 6w) | 66 | <0.0001 | 1.23(0.66, 1.79) |
| Riemersma-van (2.5mg, 6mo) | 72 | <0.0001 | 1.11(0.54, 1.67) |
| Riemersma-van (2.5mg, 1y) | 65 | <0.0001 | 1.25(0.68, 1.82) |
| Riemersma-van (2.5mg, 1.5y) | 66 | <0.0001 | 1.23(0.66, 1.80) |
| Riemersma-van (2.5mg, 2y) | 65 | <0.0001 | 1.27(0.70, 1.85) |
|  |  |  |  |
| **Gao (2.9mg, 24w)** | | | |
| Riemersma-van (2.5mg, 6w) | 65 | <0.0001 | 1.25(0.69, 1.81) |
| Riemersma-van (2.5mg, 6mo) | 72 | <0.0001 | 1.13(0.57, 1.70) |
| Riemersma-van (2.5mg, 1y) | 65 | <0.0001 | 1.28(0.70, 1.85) |
| Riemersma-van (2.5mg, 1.5y) | 66 | <0.0001 | 1.25(0.68, 1.82) |
| Riemersma-van (2.5mg, 2y) | 65 | <0.0001 | 1.30(0.72, 1.87) |

**Remarks:** The row with the author’s name and year indicated the result by included of the author’s study in meta-analysis.

**Supplementary S-Table 7.2 (Data of Figure 6). Change in cognitive functions at different dosages (MMSE).**

| **Author, year** | **I^2^** | **P-value** | **Meta-analysis Results** |
| --- | --- | --- | --- |
| Original | 0 | 0.20 | 0.49 (-0.26, 1.25) |
| *Less than or equal to 4.9mg* |  |  |  |
| **Gao (2.9mg, 12w)** | | | |
| Riemersma-van (2.5mg, 6w) | 0 | 0.20 | 0.60 (-0.31, 1.51) |
| Riemersma-van (2.5mg, 6mo) | 10 | 0.57 | 0.27 (-0.65, 1.19) |
| Riemersma-van (2.5mg, 1y) | 0 | 0.21 | 0.61 (-0.34, 1.57) |
| Riemersma-van (2.5mg, 1.5y) | 0 | 0.24 | 0.56 (-0.38, 1.50) |
| Riemersma-van (2.5mg, 2y) | 0 | 0.18 | 0.66 (-0.31, 1.62) |
|  |  |  |  |
| **Gao (2.9mg, 24w)** | | | |
| Riemersma-van (2.5mg, 6w) | 0 | 0.16 | 0.66 (-0.25, 1.57) |
| Riemersma-van (2.5mg, 6mo) | 17 | 0.48 | 0.33 (-0.59, 1.25) |
| Riemersma-van (2.5mg, 1y) | 0 | 0.16 | 0.68 (-0.28, 1.63) |
| Riemersma-van (2.5mg, 1.5y) | 0 | 0.20 | 0.62 (-0.32, 1.57) |
| Riemersma-van (2.5mg, 2y) | 0 | 0.14 | 0.72 (-0.24, 1.69) |

**Remarks:** The row with the author’s name and year indicated the result by included of the author’s study in meta-analysis.

**Supplementary S-Table 7.3 (Data of Figure 8). Change in cognitive functions across different types of cognitive impairment (MMSE).**

| **Author, year** | **I^2^** | **P-value** | **Meta-analysis Results** |
| --- | --- | --- | --- |
|  | 0 | 0.83 | 0.09 (-0.75, 0.94) |
| *Alzheimer’s dementia* |  |  |  |
| Gao (2.9mg, 12w) | 0 | 0.93 | 0.04 (-0.84, 0.92) |
| Gao (2.9mg, 24w) | 0 | 0.97 | -0.02 (-0.90, 0.86) |
|  |  |  |  |
|  | 0 | 0.36 | 0.46 (-0.53, 1.46) |
| Mixed or other types | | | |
| Riemersma-van (2.5mg, 6w) | 0 | 0.22 | 0.83 (-0.49, 2.15) |
| Riemersma-van (2.5mg, 6mo) | 48 | 0.85 | 0.13 (-1.22, 1.49) |
| Riemersma-van (2.5mg, 1y) | 0 | 0.22 | 0.92 (-0.55, 2.38) |
| Riemersma-van (2.5mg, 1.5y) | 0 | 0.28 | 0.78 (-0.64, 2.20) |
| Riemersma-van (2.5mg, 2y) | 0 | 0.17 | 1.04 (-0.46, 2.53) |

**Remarks:** The row with the author’s name and year indicated the result by included of the author’s study in meta-analysis.

**Supplementary S-Table 7.4 (Data of Figure 9). Change in cognitive functions across different levels of dementia (MMSE).**

| **Author, year** | **I^2^** | **P-value** | **Meta-analysis Results** |
| --- | --- | --- | --- |
| Original | 0 | 0.09 | 1.32 (-0.20, 2.84) |
| *Mild level* |  |  |  |
| Gao (2.9mg, 12w) | 0 | 0.14 | 1.33 (-0.45, 3.11) |
| Gao (2.9mg, 24w) | 0 | 0.09 | 1.55 (-0.22, 3.33) |
|  |  |  |  |
| Original | 0 | 0.97 | 0.01 (-0.69, 0.72) |
| *Moderate level* |  |  |  |
| Riemersma-van (2.5mg, 6w) | 0 | 0.96 | 0.02 (-0.78, 0.83) |
| Riemersma-van (2.5mg, 6mo) | 0 | 0.56 | -0.24 (-1.05, 0.57) |
| Riemersma-van (2.5mg, 1y) | 0 | 0.98 | -0.01 (-0.84, 0.82) |
| Riemersma-van (2.5mg, 1.5y) | 0 | 0.93 | -0.04 (-0.86, 0.79) |
| Riemersma-van (2.5mg, 2y) | 0 | 0.97 | 0.02 (-0.82, 0.85) |

**Remarks:** The row with the author’s name and year indicated the result by included of the author’s study in meta-analysis.

**Supplementary S-Table 8. PRISMA checklist 2020**

| **Section and Topic** | **Item #** | **Checklist item** | **Location where item is reported** |
| --- | --- | --- | --- |
| **TITLE** | | |  |
| Title | 1 | Identify the report as a systematic review. | Page 3 |
| **ABSTRACT** | | |  |
| Abstract | 2 | See the PRISMA 2020 for Abstracts checklist. | Page 2 |
| **INTRODUCTION** | | |  |
| Rationale | 3 | Describe the rationale for the review in the context of existing knowledge. | Page 3 - 5 |
| Objectives | 4 | Provide an explicit statement of the objective(s) or question(s) the review addresses. | Page 5 |
| **METHODS** | | |  |
| Eligibility criteria | 5 | Specify the inclusion and exclusion criteria for the review and how studies were grouped for the syntheses. | Page 5 - 6 |
| Information sources | 6 | Specify all databases, registers, websites, organisations, reference lists and other sources searched or consulted to identify studies. Specify the date when each source was last searched or consulted. | Page 6 - 7 |
| Search strategy | 7 | Present the full search strategies for all databases, registers and websites, including any filters and limits used. | Page 6 - 7, Supplementary S-Table 1 |
| Selection process | 8 | Specify the methods used to decide whether a study met the inclusion criteria of the review, including how many reviewers screened each record and each report retrieved, whether they worked independently, and if applicable, details of automation tools used in the process. | Page 7 |
| Data collection process | 9 | Specify the methods used to collect data from reports, including how many reviewers collected data from each report, whether they worked independently, any processes for obtaining or confirming data from study investigators, and if applicable, details of automation tools used in the process. | Page 7 - 8 |
| Data items | 10a | List and define all outcomes for which data were sought. Specify whether all results that were compatible with each outcome domain in each study were sought (e.g. for all measures, time points, analyses), and if not, the methods used to decide which results to collect. | Page 6 - 8 |
|  | 10b | List and define all other variables for which data were sought (e.g. participant and intervention characteristics, funding sources). Describe any assumptions made about any missing or unclear information. | Page 6 - 8 |
| Study risk of bias assessment | 11 | Specify the methods used to assess risk of bias in the included studies, including details of the tool(s) used, how many reviewers assessed each study and whether they worked independently, and if applicable, details of automation tools used in the process. | Page 7 - 8 |
| Effect measures | 12 | Specify for each outcome the effect measure(s) (e.g. risk ratio, mean difference) used in the synthesis or presentation of results. | Page 8 - 9 |
| Synthesis methods | 13a | Describe the processes used to decide which studies were eligible for each synthesis (e.g. tabulating the study intervention characteristics and comparing against the planned groups for each synthesis (item #5)). | Page 8 - 9 |
|  | 13b | Describe any methods required to prepare the data for presentation or synthesis, such as handling of missing summary statistics, or data conversions. | Page 8 - 9 |
|  | 13c | Describe any methods used to tabulate or visually display results of individual studies and syntheses. | Page 8 - 9 |
|  | 13d | Describe any methods used to synthesize results and provide a rationale for the choice(s). If meta-analysis was performed, describe the model(s), method(s) to identify the presence and extent of statistical heterogeneity, and software package(s) used. | Page 8 - 9 |
|  | 13e | Describe any methods used to explore possible causes of heterogeneity among study results (e.g. subgroup analysis, meta-regression). | Page 8 - 9 |
|  | 13f | Describe any sensitivity analyses conducted to assess robustness of the synthesized results. | Page 9 |
| Reporting bias assessment | 14 | Describe any methods used to assess risk of bias due to missing results in a synthesis (arising from reporting biases). | Page 7 - 8 |
| Certainty assessment | 15 | Describe any methods used to assess certainty (or confidence) in the body of evidence for an outcome. | Page 8 - 9 |
| **RESULTS** | | |  |
| Study selection | 16a | Describe the results of the search and selection process, from the number of records identified in the search to the number of studies included in the review, ideally using a flow diagram. | Page 10 – 11, Figure 1 |
|  | 16b | Cite studies that might appear to meet the inclusion criteria, but which were excluded, and explain why they were excluded. | Supplementary S-Table 3 |
| Study characteristics | 17 | Cite each included study and present its characteristics. | Page 10 - 11 |
| Risk of bias in studies | 18 | Present assessments of risk of bias for each included study. | Page 11 - 12, Supplementary S-Table 4 |
| Results of individual studies | 19 | For all outcomes, present, for each study: (a) summary statistics for each group (where appropriate) and (b) an effect estimate and its precision (e.g. confidence/credible interval), ideally using structured tables or plots. | Page 12 – 15, Table 1 |
| Results of syntheses | 20a | For each synthesis, briefly summarise the characteristics and risk of bias among contributing studies. | Page 11 – 15 |
|  | 20b | Present results of all statistical syntheses conducted. If meta-analysis was done, present for each the summary estimate and its precision (e.g. confidence/credible interval) and measures of statistical heterogeneity. If comparing groups, describe the direction of the effect. | Page 11 – 15, Figure 1 - 10 |
|  | 20c | Present results of all investigations of possible causes of heterogeneity among study results. | Page 11 – 15 |
|  | 20d | Present results of all sensitivity analyses conducted to assess the robustness of the synthesized results. | Page 15, Supplementary S-Table 5 |
| Reporting biases | 21 | Present assessments of risk of bias due to missing results (arising from reporting biases) for each synthesis assessed. | Page 11 - 12 |
| Certainty of evidence | 22 | Present assessments of certainty (or confidence) in the body of evidence for each outcome assessed. | Page 11 - 12 |
| **DISCUSSION** | | |  |
| Discussion | 23a | Provide a general interpretation of the results in the context of other evidence. | Page 16 - 18 |
|  | 23b | Discuss any limitations of the evidence included in the review. | Page 19 - 20 |
|  | 23c | Discuss any limitations of the review processes used. | Page 19 - 20 |
|  | 23d | Discuss implications of the results for practice, policy, and future research. | Page 20 |
| **OTHER INFORMATION** | | |  |
| Registration and protocol | 24a | Provide registration information for the review, including register name and registration number, or state that the review was not registered. | Page 5 |
|  | 24b | Indicate where the review protocol can be accessed, or state that a protocol was not prepared. | Page 5 |
|  | 24c | Describe and explain any amendments to information provided at registration or in the protocol. | Page 9 |
| Support | 25 | Describe sources of financial or non-financial support for the review, and the role of the funders or sponsors in the review. | Page 22 |
| Competing interests | 26 | Declare any competing interests of review authors. | Page 22 |
| Availability of data, code and other materials | 27 | Report which of the following are publicly available and where they can be found: template data collection forms; data extracted from included studies; data used for all analyses; analytic code; any other materials used in the review. | Page 22 |

*From:*  Page MJ, McKenzie JE, Bossuyt PM, Boutron I, Hoffmann TC, Mulrow CD, et al. The PRISMA 2020 statement: an updated guideline for reporting systematic reviews. BMJ 2021;372:n71. doi: 10.1136/bmj.n71

**Supplementary S-Table 9. PRISMA Abstract checklist 2020**

| **Section and Topic** | **Item #** | **Checklist item** | **Reported (Yes/No)** |
| --- | --- | --- | --- |
| **TITLE** | | |  |
| Title | 1 | Identify the report as a systematic review. | Yes |
| **BACKGROUND** | | |  |
| Objectives | 2 | Provide an explicit statement of the main objective(s) or question(s) the review addresses. | Yes |
| **METHODS** | | |  |
| Eligibility criteria | 3 | Specify the inclusion and exclusion criteria for the review. | Yes |
| Information sources | 4 | Specify the information sources (e.g. databases, registers) used to identify studies and the date when each was last searched.` | Yes |
| Risk of bias | 5 | Specify the methods used to assess risk of bias in the included studies. | Yes |
| Synthesis of results | 6 | Specify the methods used to present and synthesise results. | Yes |
| **RESULTS** | | |  |
| Included studies | 7 | Give the total number of included studies and participants and summarise relevant characteristics of studies. | Yes |
| Synthesis of results | 8 | Present results for main outcomes, preferably indicating the number of included studies and participants for each. If meta-analysis was done, report the summary estimate and confidence/credible interval. If comparing groups, indicate the direction of the effect (i.e. which group is favoured). | Yes |
| **DISCUSSION** | | |  |
| Limitations of evidence | 9 | Provide a brief summary of the limitations of the evidence included in the review (e.g. study risk of bias, inconsistency and imprecision). | Yes |
| Interpretation | 10 | Provide a general interpretation of the results and important implications. | Yes |
| **OTHER** | | |  |
| Funding | 11 | Specify the primary source of funding for the review. | Yes |
| Registration | 12 | Provide the register name and registration number. | Yes |

*From:*  Page MJ, McKenzie JE, Bossuyt PM, Boutron I, Hoffmann TC, Mulrow CD, et al. The PRISMA 2020 statement: an updated guideline for reporting systematic reviews. BMJ 2021;372:n71. doi: 10.1136/bmj.n71
